# Reaction Mechanisms in Copper Atomic Layer Deposition Using Copper(II) Hexafluoroacetylacetonate and Diethylzinc *via* *In Situ* Time-of-Flight Mass Spectrometry

**DOI:** 10.1021/acs.chemmater.5c01521

**Published:** 2025-09-11

**Authors:** Camilla Minzoni, Sylwia Klejna, Krzysztof Mackosz, Caroline Hain, Aleksandra Figura-Jagoda, Aleksandra Szkudlarek, Marcin Sikora, Andreas Werbrouck, Ramakrishna Ramisetty, Patrik Hoffmann, Ivo Utke

**Affiliations:** † 34314 Empa, Swiss Federal Laboratories for Materials Science and Technology, Feuerwerkerstrasse 39, 3602 Thun, Switzerland; ‡ EPFL, École Polytechnique Fédérale de Lausanne, Station 17, 1015 Ecublens, Switzerland; § 49811 AGH University of Krakow, Academic Centre for Materials and Nanotechnology, Al. Mickiewicza 30, 30-059 Krakow, Poland; ∥ National Synchrotron Radiation Centre SOLARIS, Jagiellonian University, Czerwone Maki 98, 30-392 Krakow, Poland; ⊥ Materials Science and Engineering Institute, University of Missouri, Laferre Hall, 416 S Sixth St, Columbia, Missouri 65201, United States; # 375013 TOFWERK AG, Schorenstrasse 39, 3645 Thun, Switzerland

## Abstract

The development of high-quality copper (Cu) thin films by atomic layer deposition (ALD) remains challenging due to a limited understanding of the reaction mechanisms that govern film growth and composition. To address this gap, in this study, time-of-flight mass spectrometry (TOFMS) was used to perform the first comprehensive *in situ* investigation of the surface reactions during Cu ALD, using dehydrated Cu­(II) hexafluoroacetylacetonate (Cu­(hfac)_2_) (CAS 14781–45–4) and diethylzinc (DEZ, ZnEt_2_) (CAS 557–20–0). The high sensitivity and mass resolution of TOFMS enabled the identification of previously only hypothesized surface reaction volatile by-products, revealing two different mechanisms in each ALD half-cycle. During the Cu­(hfac)_2_ pulse, both Zn­(hfac)_2_ and EtZn­(hfac) volatile by-products were detected in varying amounts, while the DEZ pulse predominantly yielded EtZn­(hfac). X-ray absorption spectroscopy (XAS) of the as-deposited films confirmed the formation of metallic copper with minimal zinc (Zn) and carbon (C) incorporation. The results underline simultaneous full and half-ligand exchange mechanisms during the precursor pulse, resulting in a surface saturated with hfac ligands. However, the reaction mostly happens in the DEZ half-cycle, which is dominated by a partial ligand exchange pathway, leading to the saturation surface being composed of Zn species. This study provides the first experimental evidence of the mechanistic pathways for copper deposition and demonstrates the value of *in situ* TOFMS for monitoring ALD reaction mechanisms, a critical aspect for process development and optimization.

## Graphical abstract

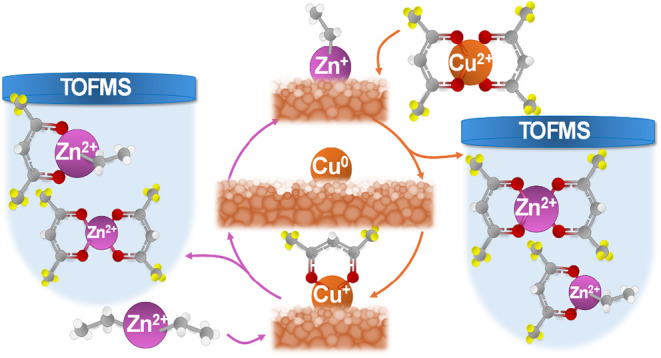

## 1 Introduction

The fabrication of copper (Cu) thin films is a highly sought-after process with applications across a range of fields. These include micro- and nanoelectronics (*e.g.*, interconnects),^1,2^ flexible electronic devices,^3^ and catalysis applications.^4^ Nevertheless, achieving precise control over the Cu deposition processes to obtain the desired quality has proven challenging. Atomic layer deposition (ALD) has emerged as a promising technique, offering the ability to produce conformal, uniform films with atomic-level thickness control through a cyclic, self-limiting surface process. However, despite extensive studies of ALD reaction mechanisms in metal oxides,^5–10^ the fundamental understanding of metal ALD remains limited by a lack of *in situ* experimental investigations.^11,12^ The development of metal ALD processes faces significant challenges, primarily due to an incomplete understanding of the underlying surface reaction mechanisms. Metal ALD typically exhibits 3D island growth mode, with submonolayer surface coverages often below 10% in the initial stages of the deposition.^13^ This behavior can be attributed to multiple factors: (i) steric hindrance effects from the metalorganic precursors ligands upon chemisorption,^13^ (ii) disabled/or hampered nucleation caused by ligands or contaminants adsorption on the substrate functional groups, preventing further precursor chemisorption^13^ (iii) difficulties in identifying suitable co-reactants that can effectively chemically reduce metal precursors at temperatures as low as 100 °C. These conditions are ideal for minimizing thermally activated metal atom mobility and mitigating dewetting, which is often driven by surface energy mismatches between the metal film and the substrate.^14–16^ These challenges result in discontinuous and rougher films compared to the films grown by layer-by-layer. Therefore, direct *in situ* measurements of surface reactions are essential for understanding and controlling the growth mechanism during metal ALD processes and, more specifically, copper deposition.

In recent years, several Cu ALD processes have been developed employing a variety of Cu­(I) and Cu­(II) organometallic compounds and co-reagents. Notably, Karppinen et al.^17^ developed a low-temperature (160–240 °C) ALD process using an organic reducing agent. This process, based on the sequential pulsing of copper­(II) acetylacetonate (Cu­(acac)_2_); (Cu­(C_5_H_7_O_2_)_2_, CAS 13395–16–9) and hydroquinone (HQ; C_6_H_4_(OH)_2_, CAS 123–31–9), yielded highly crystalline copper metal films with a growth rate of 1.8 Å/cycle. Significant improvements in Cu ALD were made by Lee et al.;^18^ they demonstrated Cu film growth at temperatures as low as 100–120 °C using a novel approach that combined diethylzinc (DEZ; ZnEt_2_; Zn­(C_2_H5)_2_) as the reducing agent with copper­(II) dimethylamino-2-propoxide (Cu­(dmap)_2_; Cu­(C_5_H_12_NO)_2_, CAS 185827–91–2). This process in its steady-state regime is hypothesized to operate *via* a transmetalation reaction, a two-step mechanism. In the initial stage, a ligand exchange reaction occurs between the copper complex and DEZ, generating volatile Zn­(dmap)_2_ (Zn­(C_5_H_12_NO)_2_) and leaving copper alkyl species on the surface. These intermediates then undergo rapid reductive elimination, yielding gaseous butane (C_4_H_10_) and metallic copper as the final layer.^19^ A similar ALD process was later reported by Zhong et al.,^20^ using DEZ with copper­(II) hexafluoroacetylacetonate (Cu­(hfac)_2_; Cu­(C_5_HF_6_O_2_)_2_) as the copper precursor. In this case, the ALD window was relatively narrow, ranging from 180 to 200 °C. Recently, Soltani et al.^21^ also fabricated Cu thin films for battery applications using the same reactants. However, these approaches led to the incorporation of Zn into the deposited film, which, on one hand, can affect its suitability for microelectronics among other applications, but on the other hand, can enhance catalytic^22^ and antimicrobial properties.^23^ Therefore, understanding the reaction mechanisms and resulting surface chemistry is essential for controlling Zn incorporation and tailoring the material properties to specific applications. The presence of Zn in the film may arise from multiple pathways, such as from the reaction of residual moisture (H_2_O) with DEZ, leading to the formation of zinc oxide,^24^ and the decomposition of not desorbed by-products (*i.e.*, ZnL_2_ with L = hfac, dmap). Additionally, DEZ molecules may undergo β-hydride elimination, a decomposition reaction that could lead to a direct metallic zinc deposition. While this reaction has been experimentally confirmed in the gas phase^25^ and on SiO_2_, Pd, and Rh surfaces,^26–28^ it has not been conclusively observed under ALD conditions. Nevertheless, the presence of metallic zinc impurities in the films deposited by Lee and Zhong,^18,20^ suggests that this reaction may indeed occur during the ALD cycles.

To gain insight into the reaction mechanism, Vidjayacoumar et al.^29^ investigated the possibility of this ligand exchange reaction in Cu ALD by conducting solution-based copper deposition followed by nuclear magnetic resonance (NMR) screening. A comprehensive range of copper complexes, including Cu­(hfac)_2_, was examined along with three different organometallic reducing agents, namely, trimethylaluminum (TMA), triethylborane (TEB), and diethylzinc (DEZ). The presence of intermediates, including LMEt (M = Cu or Zn, L = ligand, Et = ethyl) and LEt, was determined, along with the volatile by-products ML_2_ and Et_2_ (M = Zn, L = ligand, Et = ethyl). Dey and Elliott^30,31^ applied DFT calculations to assess the thermodynamics of transmetalation for an array of precursors, including Cu­(acac)_2_, Cu­(dmap)_2_, and Cu­(hfac)_2_, investigating the effect of various ligands in steady-state ALD. The studied reaction steps were disproportionation, ligand exchange, and reductive elimination. In particular, two possible pathways were considered: first, as initially suggested by Lee et al.,^18^ a *complete ligand exchange* ([Disp-formula eq1])­

1 $${\mathrm{CuL}}_{2\left(g\right)}+{\mathrm{ZnEt}}_{2\left(g\right)}\to s‐\mathrm{Cu}\left(0\right)+{\mathrm{ZnL}}_{2\left(g\right)}+{\mathrm{Et}}_{2\left(g\right)}$$

with half Reaction 1-1 of the metal precursor pulse and (1-2) of the DEZ pulse

1-1 $$s‐\mathrm{Zn}‐\mathrm{Et}+s‐\mathrm{Cu}‐\mathrm{Et}+2{\mathrm{CuL}}_{2\left(g\right)}\to 2s‐\mathrm{Cu}‐L+s‐\mathrm{Cu}\left(0\right)+{\mathrm{ZnL}}_{2\left(g\right)}+{\mathrm{Et}}_{2\left(g\right)}$$

1-2 $$2s‐\mathrm{Cu}‐L+2{\mathrm{ZnEt}}_{2\left(g\right)}\to s‐\mathrm{Zn}‐\mathrm{Et}+s‐\mathrm{Cu}‐\mathrm{Et}+s‐\mathrm{Cu}\left(0\right)+{\mathrm{ZnL}}_{2\left(g\right)}+{\mathrm{Et}}_{2\left(g\right)}$$

where surface species are labeled with “s-” and gaseous molecules with “(g)”. A second considered reaction was a *partial ligand exchange* ([Disp-formula eq4]), as first observed in solution by Vidjayacoumar et al.^29^

2 $${\mathrm{CuL}}_{2\left(g\right)}+{\mathrm{ZnEt}}_{2\left(g\right)}\to s‐\mathrm{Cu}\left(0\right)+{\mathrm{EtZnL}}_{\left(g\right)}+{\mathrm{EtL}}_{\left(g\right)}$$

with half Reaction 2-1 of the metal precursor pulse and (2-2) of the DEZ pulse

2-1 $$s‐\mathrm{Zn}‐\mathrm{Et}+s‐\mathrm{Cu}‐\mathrm{Et}+2{\mathrm{CuL}}_{2\left(g\right)}\to 2s‐\mathrm{Cu}‐L+s‐\mathrm{Cu}\left(0\right)+{\mathrm{EtZnL}}_{\left(g\right)}+{\mathrm{EtL}}_{\left(g\right)}$$

2-2 $$2s‐\mathrm{Cu}‐L+2{\mathrm{ZnEt}}_{2\left(g\right)}\to s‐\mathrm{Zn}‐\mathrm{Et}+s‐\mathrm{Cu}‐\mathrm{Et}+s‐\mathrm{Cu}\left(0\right)+{\mathrm{EtZnL}}_{\left(g\right)}+{\mathrm{EtL}}_{\left(g\right)}$$

Notably, both reactions ultimately lead to the deposition of metallic copper, the formation of volatile by-products, and the same surface-saturating species: s-CuL as the product of the Cu precursor pulse, and s-ZnEt + s-CuEt remaining on the surface at the end of the DEZ pulse. Maimaiti and Elliott^32,33^ further refined the DFT mechanistic study, investigating the kinetics and surface reaction mechanisms of Cu ALD from Cu­(dmap)_2_ and ZnEt_2_ on Cu(111) surface. Two competing pathways were identified for the reaction of ZnEt_2_ with chemisorbed Cu­(dmap)_2_ molecules: (i) initial formation and desorption of butane, facilitated by ligand interactions, followed by ligands diffusion and reordering around Zn to form Zn­(dmap)_2_; and (ii) formation and desorption of Zn­(dmap)_2_ in the presence of ethyl groups with subsequent ethyl groups migration leading to butane formation. These results demonstrate the cooperative role of neighboring ligands in reducing activation barriers for by-product formation and desorption.

To date, the surface reaction volatile by-products formed during Cu ALD have not been confirmed through direct experimental measurements. Time-of-flight mass spectrometry (TOFMS) is a powerful tool for addressing this issue, providing direct insights into the gas species formed in the deposition chamber and thereby offering useful information on the surface reactions. In this context, TOFMS presents certain analytical advantages over traditional quadrupole mass spectrometry (QMS), including a broader detectable mass-to-charge range (simultaneous detection of a wide range of masses, unlike sequential scanning in QMS), higher sensitivity and resolution, and more rapid data acquisition. Its capacity for real-time measurement further enables continuous monitoring of precursor delivery, supporting evaluations of process stability and repeatability—important factors for achieving controlled and optimized deposition conditions.^34^ Building upon the findings of Priebe et al.,^34^ who demonstrated the successful application of TOFMS for *in situ* monitoring of alumina ALD, we extended this analytical approach to the deposition of metals.

To the best of the authors’ knowledge, this study is the first to experimentally monitor the reaction mechanisms of Cu ALD under ALD conditions. Initially, a Cu thin film was deposited using dehydrated Cu­(hfac)_2_ and DEZ under the optimized conditions to minimize Zn contamination. The deposited Cu film was characterized using X-ray absorption spectroscopy (XAS) to determine its chemical composition, while scanning electron microscopy (SEM) and atomic force microscopy (AFM) provided information on morphology, conformality, and surface roughness. Subsequently, we conducted the first in-depth experimental investigation of the Cu ALD reaction mechanisms using *in situ* TOFMS to monitor volatile surface reaction by-products. Minor and unexpected species were identified and analyzed, providing robust evidence of the Cu ALD mechanism. The findings confirm previously unverified chemical pathways and surface compositions at saturation, offering insights into the surface chemistry of metal deposition with the use of a reducing agent.

## 2 Experimental Section

### 2.1 Materials

Copper­(II) hexafluoroacetylacetonate hydrate Cu­(hfac)_2_ *x*H_2_O (99.99% STREM, USA) (CAS 155640–85–0) was used as the source of Cu. As the presence of water molecules in the hydrated complex^14^ caused nonreproducible precursor transport, an in-house dehydration procedure was performed before each ALD process to obtain dehydrated Cu­(hfac)_2_ (CAS 14781–45–4). (See Supporting Information S1.2 for a detailed dehydration process). Diethylzinc ZnEt_2_ (EG Electronic grade, Dock Chemicals, Germany) (CAS 557–20–0) was selected as the reducing agent. High-purity argon (Ar) (99.9995%, Air Liquide, France) was used as a carrier and purging gas.

### 2.2 Methods

#### 2.2.1 ALD Process

All processes were carried out in a custom-built hot-wall ALD chamber equipped with an XDS35i dry scroll pump (Edwards Vacuum, U.K.). The pump operated at a speed of 35 m^3^/h, maintaining a background pressure of approximately 2 × 10^–2^ mbar. A Cu film was deposited onto a Si (100) substrate with a native SiO_2_ layer (MicroChemicals, Germany). The deposition process consisted of 650 ALD cycles, with each cycle involving a 300 ms pulse of Cu­(hfac)_2_ and a 50 ms pulse of DEZ, separated by 5 min Ar purge intervals at 100 cm^3^/min (sccm). Cu­(hfac)_2_ was delivered using an exposure mode configuration, in which the exhaust valve was closed 500 ms prior to the precursor pulse (p_chamber_ = 2.3 × 10^–2^ mbar at 0 sccm Ar flow). The Ar flow was set to 15 sccm, 1 s after the precursor pulse. The system then remained in this state for an additional 1 s, resulting in a total exposure time of 2 s (p_chamber_ raised to final 6.3 × 10^–2^ mbar). Subsequently, the exhaust valve was reopened for the purge step. In contrast, DEZ was delivered using a continuous flow approach, with the exhaust valve remaining open and at a constant Ar flow of 20 sccm throughout the entire DEZ half-cycle (p_chamber_ = 1.3 × 10^–1^ mbar). For subsequent TOFMS monitoring, the process was modified to use the exposure mode also during the reducing agent half-cycle, enabling better tracking of the gas-phase species throughout the ALD reaction. Specifically, the exhaust valve was closed 100 ms prior to the DEZ pulse (maximum p_chamber_ = 5.9 × 10^–1^ mbar at 20 sccm Ar flow, exhaust valve closed). After the pulse, the system remained in this state for 2 s, and then the exhaust valve was reopened for the purge step. The precursor reservoirs for Cu­(hfac)_2_ and DEZ were maintained at 80 and 5 °C, respectively. The DEZ reservoir was kept at 5 °C for reducing its volatility, thereby minimizing any possible incorporation of zinc impurities into the layer. The substrate temperature was held at 190 °C throughout the process, Ar lines were kept at 100 °C, while the exhaust valve and the chamber walls were both at 120 °C. Detailed process parameters are provided in Table S1 of Supporting Information S1.

#### 2.2.2 Cu Film Characterization

The chemical and phase composition of the deposited film was examined using X-ray absorption spectroscopy (XAS). XAS measurements were performed at the PIRX beamline of the SOLARIS National Synchrotron Radiation Centre (Kraków, Poland).^35^ Spectra were collected at room temperature from a sample kept at ultrahigh vacuum (*p* < 10^–10^ mbar) using volume-sensitive fluorescence (TFY) and surface-sensitive total electron yield (TEY) detection schemes. The former was probed using an Amptek FastSDD X-ray Detector equipped with a C2 window (Si_3_N_4_ coated with Al). Total fluorescence yield was probed by integration of the photons in the energy window from approximately 200 eV to approximately 2000 eV, while Cu Lα partial fluorescence yield was probed by integration of the photons in the energy window from approximately 850 eV to approximately 950 eV. Total electron yield was probed as the drain current of an unbiased sample. Photon beam extracted from the bending magnet was monochromatized with high resolving power (*E*/Δ*E* > 2000) using 800 lines/mm linear plan grating and exit slit opened to 50 μm. A survey scan across a wide energy range from carbon K-edge to Zn L-edge was probed to qualitatively determine the chemical composition of the film. In addition, the Cu L_3_-edge was probed with high energy resolution to resolve different Cu oxidation states, which can be observed as subtle changes in the XAS spectral shape. Speciation of Cu phases was determined *via* linear combination fitting of the Cu L_3_-edge XAS spectra using reference data of three systems: metallic Cu, Cu_2_O, and CuO. Reference spectra are plotted in Supporting Information S3, Figure S10.

Scanning electron microscopy (SEM; Tescan Mira, Czech Republic) and atomic force microscopy (AFM) provided insights into the film morphology, conformality, and surface roughness. The AFM analysis was performed using a Bruker Dimension ICON XR microscope in the Peak Force Tapping mode. The Peak Force Amplitude was set to 150 nm with the Peak Force Frequency equal to 2 kHz and a scanning rate of 0.894 Hz. The SCANASYST-AIR probe by Bruker was used with a silicon tip on a nitride lever (thickness = 650 nm, length = 115 μm, width = 25 μm). The cantilever with the dimensions of the spring constant was equal to 0.4 N/m and a resonance frequency of 70 kHz.

#### 2.2.3 *In Situ* TOFMS

The ALD process was monitored *in situ* using a TOFMS (Process Analyzer, model PA-G, TOFWERK AG, Switzerland) with a mass resolving power of *R* = *m*/Δ*m* = 4000. The TOFMS was connected directly to the deposition chamber *via* a KF25 bellows held at 100 °C. The flow of analytes into the TOFMS was controlled using a manual leak valve (EVN 116, Pfeiffer Vacuum, Germany) connected at the bellows entrance. The experimental setup is shown in Figure 1. The TOFMS system consisted of an electron ionization (EI) source (equipped with an open configuration single channel ion chamber), a notch filter, an orthogonal extraction unit, a single reflectron TOF analyzer (HTOF), and a multichannel plate (MCP) electron multiplier (Photonis, USA).

**Figure 1:**
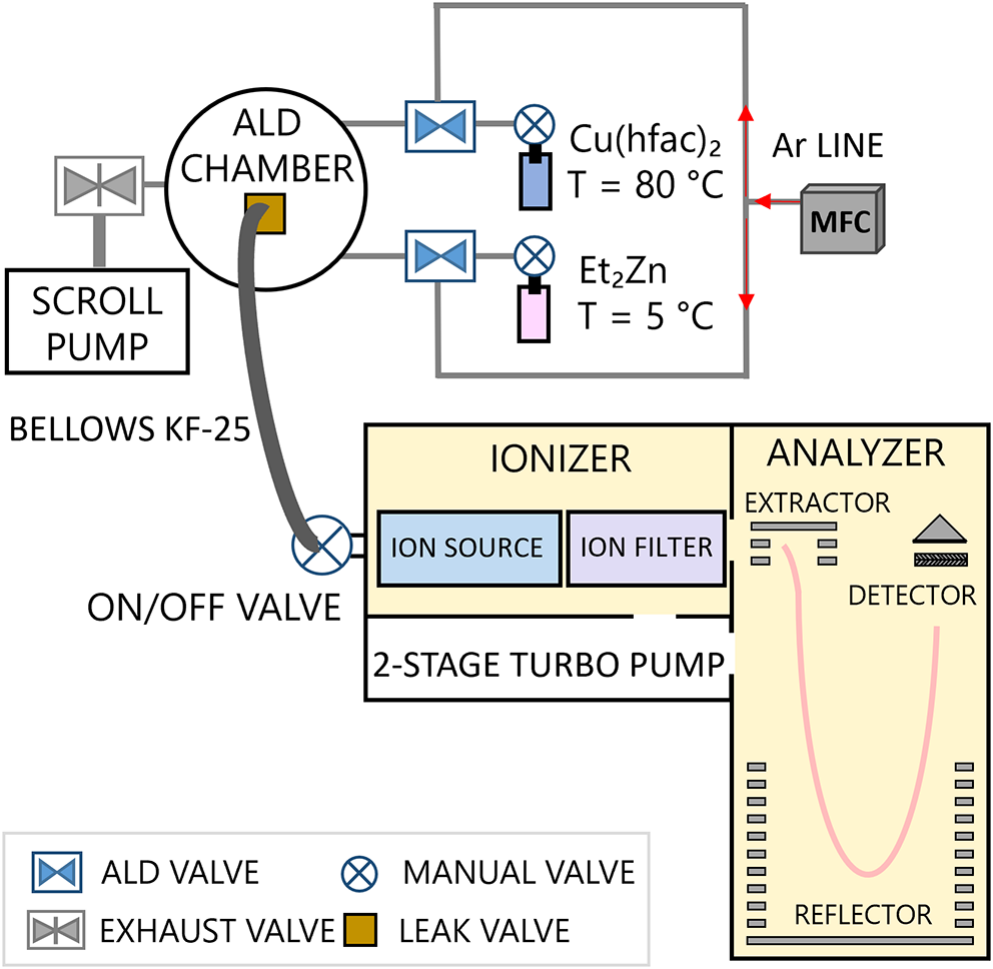
Schematic of the ALD + TOFMS experimental system. Wall and substrate temperatures were maintained at 120 and 190 °C, respectively. The exhaust was also held at 120 °C and the Ar lines were maintained at 100 °C. The TOFMS was directly connected to the ALD chamber *via* a KF25 bellows connection, heated at 100 °C. The ionizer background pressure was 2.5 × 10^–6^ mbar, while the operating pressure during *in situ* measurements ranged between 4 × 10^–6^ and 2 × 10^–4^ mbar.

The notch filter is an ion guide/filter system that attenuates high-intensity signals of less-interest molecules while directing the ionized molecules from the ion source to the TOF analyzer. The notch filter significantly enhances the effective dynamic range of the instrument, allowing peaks of lower intensity to be clearly detected and analyzed. Additionally, it reduces the risk of saturation in the MCP detector. In this experiment, the following ion signals were notched: Ar^+^ (*m*/*z* 40), Ar^2+^ (*m*/*z* 20), CF_3_^+^ (*m*/*z* 69), and DEZ M^+^ (*m*/*z* 122). The system operated with an ionization energy of 70 eV and an emission current of 0.5 mA. The monitored mass-to-charge ratio (*m*/*z*) range was set between 15 and 500 to allow the detection of parent peaks and fragments of both reactants and surface reaction volatile by-products. Accurate mass calibration across this range was ensured using a five-point calibration method; see Supporting Information S2.1. Peak assignments were performed by comparing experimental *m*/*z* ratios with the theoretical *m*/*z* values of the expected fragments. To ensure an accurate identification, this analysis was combined with isotopic distribution profiling. The distinctive isotopic patterns served as molecular fingerprints, allowing for discrimination between compounds with similar masses but different elemental compositions, see Supporting Information S2.2–S2.3.

Prior to the *in situ* TOFMS Cu ALD study, a control experiment was performed to assess the potential influence of pressure-related fluctuations on the baseline TOFMS peak signals, see Supporting Information S2.4. This experiment simulated an ALD process without the actual pulsing of the reactants (closed manual valves of the reactant reservoirs) and was already performed by Hornsveld et al.^36^ to regulate their quadrupole mass spectrometric measurements. In our *in situ* TOFMS setup, only butane showed mass signal variation with pressure, see Figure S5, simplifying the analysis of the mass spectra considerably.

## 3 Results and Discussion

### 3.1 Characterization of Cu ALD Thin Film

To assess the surface morphology of the deposited film in more detail, SEM and AFM analyses were performed. The SEM images presented in Figure 2a,b reveal a dense film, characterized by the presence of large, elongated agglomerates. Cross-section analysis confirmed good film coalescence, with an average film thickness of approximately 45 nm. The calculated growth-per-cycle (GPC) is 0.7 Å/cycle. This value falls into the reported range of GPC^17^ for Cu ALD processes. Zhong et al.^20^ for the temperature window of 180–200 °C on glass substrate observed a GPC of 1.4 Å/cycle, while Soltani et al.^21^ reported a GPC of 0.3 Å/cycle using the same reactants on silicon with a native oxide layer at 175 °C.

**Figure 2:**
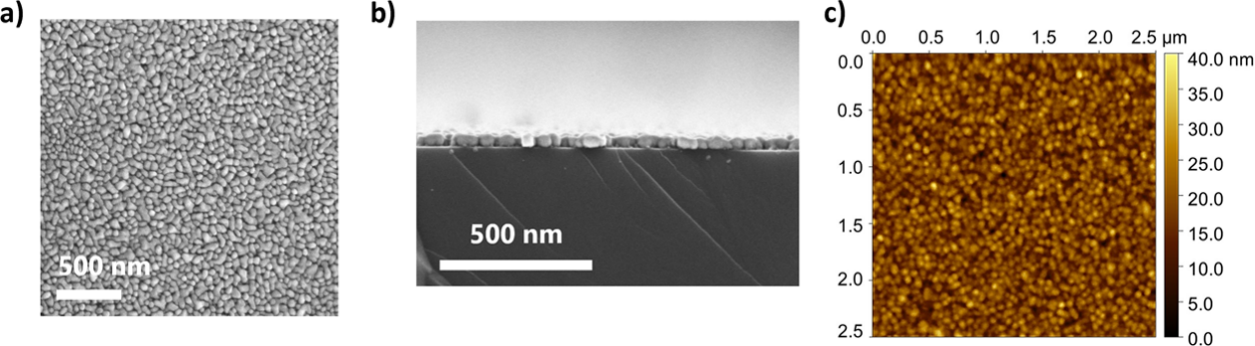
(a) SEM top-view image, (b) SEM cross-section image, and (c) AFM image of the deposited Cu ALD film.

AFM (Figure 2c) provided further insights into the topological features of the film, yielding a root-mean-square roughness of *S*_a_ = 3.95 nm and a maximum height difference of 29.3 nm, which correlates with the agglomerated microstructure observed by SEM.

Figure 3 shows the XAS spectra of the Cu thin film deposited on a Si substrate with a native oxide layer. As the L-edge absorption spectra of transition metal ions with the 3d*^n^* ground state configuration are shaped by the 2p^5^3d^*n*+1^ multiplet structure, they are considered fingerprints of the local coordination environment and oxidation state of copper and zinc. Quantitative determination of the relative volume fractions of different Cu phases present in the film was achieved through linear combination fitting of the Cu L-edge XAS, collected using Cu Lα partial fluorescence yield; see Figure 3a. The film deposited consisted mainly of metallic copper (approximately 85 ± 5 atom %), with Cu_2_O accounting for around 15 ± 5 atom %. The contribution from Cu^2+^ species, determined by using a CuO reference spectrum, was estimated to be less than 1 atom %. Given the total film thickness of 45 nm, the Cu oxide layer was approximately 4 nm thick, which is the typical thickness of a native oxide film on copper. (See Supporting Information S3 for details regarding the oxide thickness calculation). Therefore, the observed Cu^2+^ and Cu^1+^ species were probably formed during exposure of the film to air (oxygen and humidity) when transferred between the deposition and XAS chambers. Only trace amounts of Zn, estimated to be ≤1 atom %, were detected, as indicated by a weak increase in fluorescence yield over Zn L-edge (Figure 3b).

**Figure 3:**
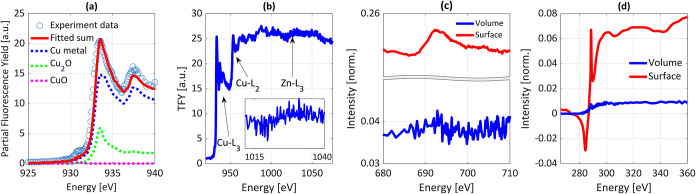
XAS analysis of the deposited Cu film on Si substrate with native oxide: (a) Linear combination fit of Cu L_3_-edge spectra collected using Lα partial fluorescence yield. (b) TFY spectra collected over a wide energy range spanning the Cu L and Zn L-edges. The Zn L-edge step (highlighted in the inset) is barely distinguishable compared to the prominent Cu L-edge. (c, d) Comparison between XAS signals from the film volume (TFY) and surface (TEY) at fluorine K-edge (c) and carbon K-edge (d). The dip observed at approximately 285 eV in the TEY spectrum at C K-edge arises from instrumental artifacts.

Furthermore, XAS collected over a wide energy range using both total fluorescence yield (TFY) and total electron yield (TEY) detection methods provided insights into the distribution of carbon and fluorine species within the film. The TFY spectrum revealed negligible presence of carbon or fluorine species in the bulk volume of the film; in contrast, the TEY spectrum, which is more surface-sensitive, showed a noticeable presence of carbon and some residues of fluorine, likely originating from residual Cu­(hfac)_2_ precursor molecules chemisorbed on the surface and carbon contamination due to air exposure. The XAS results confirm that the deposited film is primarily composed of metallic Cu, with only trace Zn contamination, demonstrating the effectiveness of the optimized parameters in achieving high-purity Cu films.

### 3.2 TOFMS Patterns

Interpreting mass spectra in the presence of multiple molecules requires identification of unique markers of the original intact (parent) molecules among numerous ionization fragments. This is especially challenging in ALD, where reactants and surface reaction volatile by-products are present simultaneously; they often produce fragments that overlap their masses.^5,34^ While ionized parent molecules are ideal identifiers, they frequently have weak or absent mass signals due to dissociative ionization being their dominant relaxation pathway upon electron impact. Table 1 presents representative *m*/*z* ratios of reactants and main isotopes of potential surface reactions, volatile by-products of full and partial ligand exchange (Reactions 1 and 4), serving as key references for further analysis.

**Table 1:**
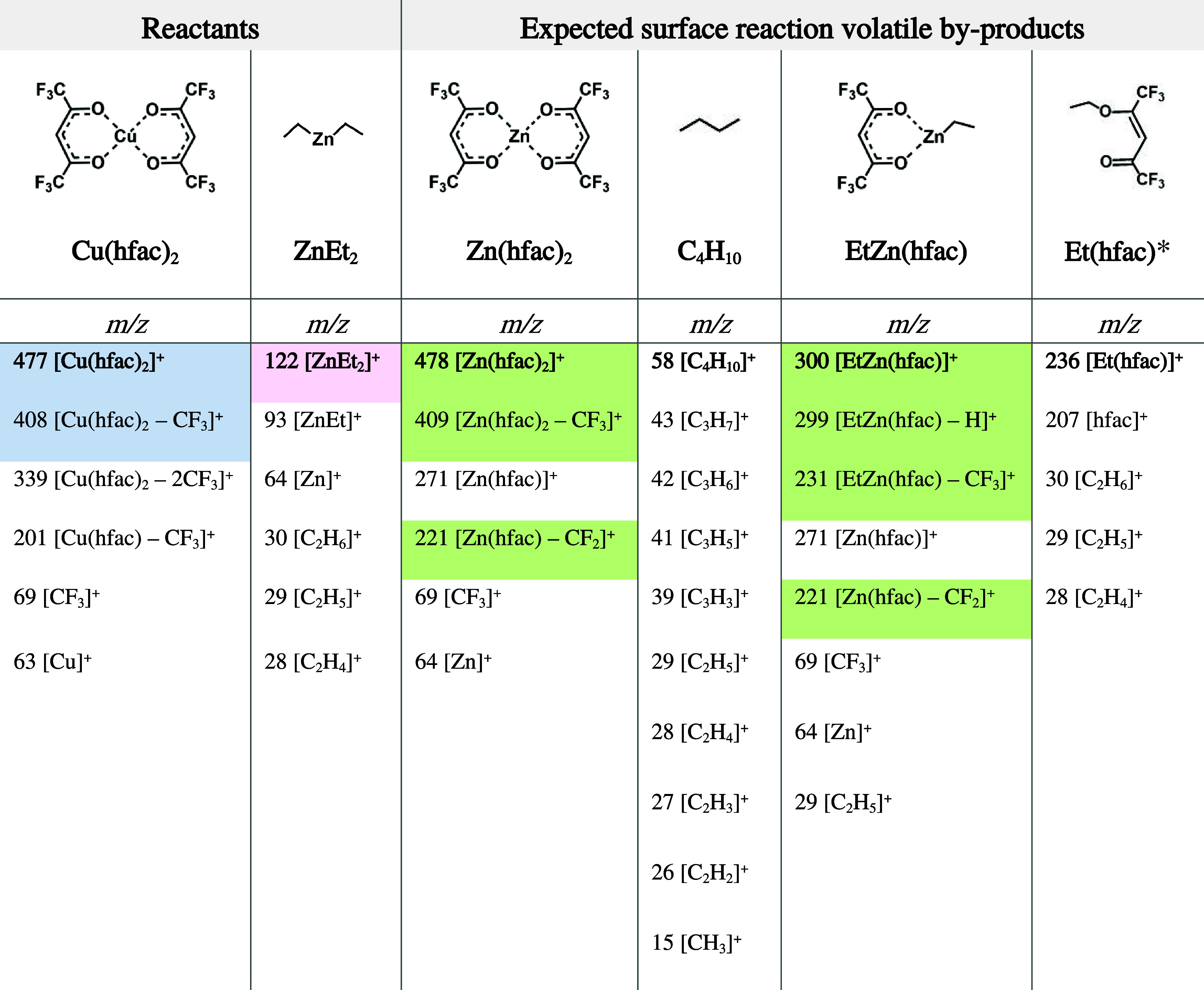
Summary of Parent Ions (bold) and Most Prominent Ionization Fragments of: Cu­(hfac)_2_, ZnEt_2_ (ALD Reactants), Zn­(hfac)_2_, EtZn­(hfac), and C_4_H_10_ (Possible Volatile Surface Reactions By-products), Sorted by *m*/*z* Values^a^ ^,^ ^b^

a Green-coded *m/z* values are unique identifiers of the surface reaction by-products, and only these peaks were used for further analysis. Light blue and pink-coded *m/z* values are unique identifiers of the reactants. See the text for explanation.

b Data reference: for Cu­(hfac)_2_,^37^ ZnEt_2_,^38^ Zn­(hfac)_2_,^39^ and C_4_H_10_.^40^ No references were found for EtZn­(hfac) nor Et­(hfac). Note that only the peaks of the most abundant isotope are reported for each compound.

* Et­(hfac) structure is hypothetical.

In this work, the MS analysis required differentiation of three structurally similar complexes: Cu­(hfac)_2_ (precursor), Zn­(hfac)_2_, and Zn­(hfac)­Et (surface reaction volatile by-products). As shown in Figure 4, the parent ions of the first two compounds differ by only one mass unit, with *m*/*z* = 477 for Cu­(hfac)_2_ and 478 for Zn­(hfac)_2_. The *m*/*z* = 1 mass difference also persists in their primary fragmentation pathway, where −CF_3_ group dissociation from Cu­(hfac)_2_ and from Zn­(hfac)_2_ yields fragments at *m*/*z* 408 and 409, respectively. The MS analysis is further complicated by the isotope peaks giving additional overlapping mass peaks at *m*/*z* 478 (+1), 479 (+2), and 480 (+3) (for Cu) and at *m*/*z* 479 (main isotope), 480 (+2) for Zn. The TOFMS system provided sufficient resolving power to achieve reliable peak separation, thereby enabling unambiguous discrimination (SI S2.3).

**Figure 4:**
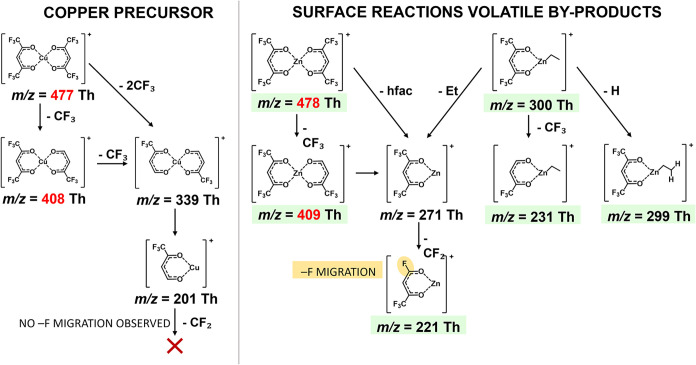
Most intense electron impact fragments of the Cu precursor (left) and Zn-containing surface reaction volatile by-products (right). Green boxes indicate unique identifiers specific to each by-product, later considered for the MS analysis, while the yellow box indicates the characteristic fluorine migration pathway observed exclusively in Zn-containing compounds. Red numbers emphasize the minimal mass difference between Cu and Zn compounds.

A key distinctive fragment for Zn-containing species results from a unique fluorine migration pathway observed exclusively in these Zn-containing species, but notably absent in Cu­(hfac)_2_,^39,41,42^ see Figure 4. This fragmentation mechanism occurs upon electron impact, resulting in the loss of a −CF_2_ group and the migration of a fluorine atom. Computational calculations^41^ indicated that the most energetically favorable site for fluorine migration is to the α-carbon; however, the fluorine may also migrate on the metallic cation. This distinctive fragment (*m*/*z* 221) provides conclusive evidence for the formation of Zn complexes and supports the proposed ligand exchange mechanism. However, it does not allow on its own to differentiate between the two possible Zn products, *i.e.*, Zn­(hfac)_2_ and EtZn­(hfac), as both exhibit this characteristic fragmentation behavior. Distinguishing between these two species relies on their different parent ions and initial fragments, as shown in Figure 4.

The spectral areas corresponding to the butane region (*m*/*z* = 58 – 16) as well as to the Et­(hfac) region (*m*/*z* = 236 – 16) present significant analytical challenges. The parent ion of Et­(hfac) was not detected, and moreover, its fragments overlap with ligands present in the reactant molecules, further complicating the analysis. The butane parent peak intensity exhibited pressure-dependent variations throughout the ALD process, as confirmed by the control experiments performed prior to the ALD cycles (SI S2.4, Figure S5). Consequently, butane and Et­(hfac) signals were excluded from further analysis; however, their formation during the ALD process cannot be ruled out.

Therefore, only the unique identifiers highlighted in green in Table 1 and Figure 4 were selected for *in situ* ALD monitoring and subsequent detailed analysis.

### 3.3 *In Situ* TOFMS Monitoring of Cu ALD Deposition

The time-resolved (TS) mass spectrum presented in Figure 5 shows the temporal evolution of the parent ion signals, measured at their exact masses for accurate identification, over four ALD cycles. Each ALD cycle consisted of alternating pulses of the Cu­(hfac)_2_ precursor and DEZ as the reducing agent. Each reactant pulse was followed by a 5 min Ar purge to ensure that the two reactants were never simultaneously present in the gas phase, as confirmed by the decay to noise level of their TOFMS signals and the absence of the simultaneous presence of Cu­(hfac)_2_ and DEZ parent peaks in the MS spectra. This temporal separation confirmed that the ALD process proceeded *via* surface reactions rather than gas phase interactions, maintaining the self-limiting nature of the process. The highest counts of the first Cu­(hfac)_2_ pulse are probably due to near-equilibrium saturation conditions in the precursor reservoir. This maximum vapor pressure was not achieved again in subsequent pulses; instead, the system reached a lower but consistent steady-state vapor pressure.

**Figure 5:**
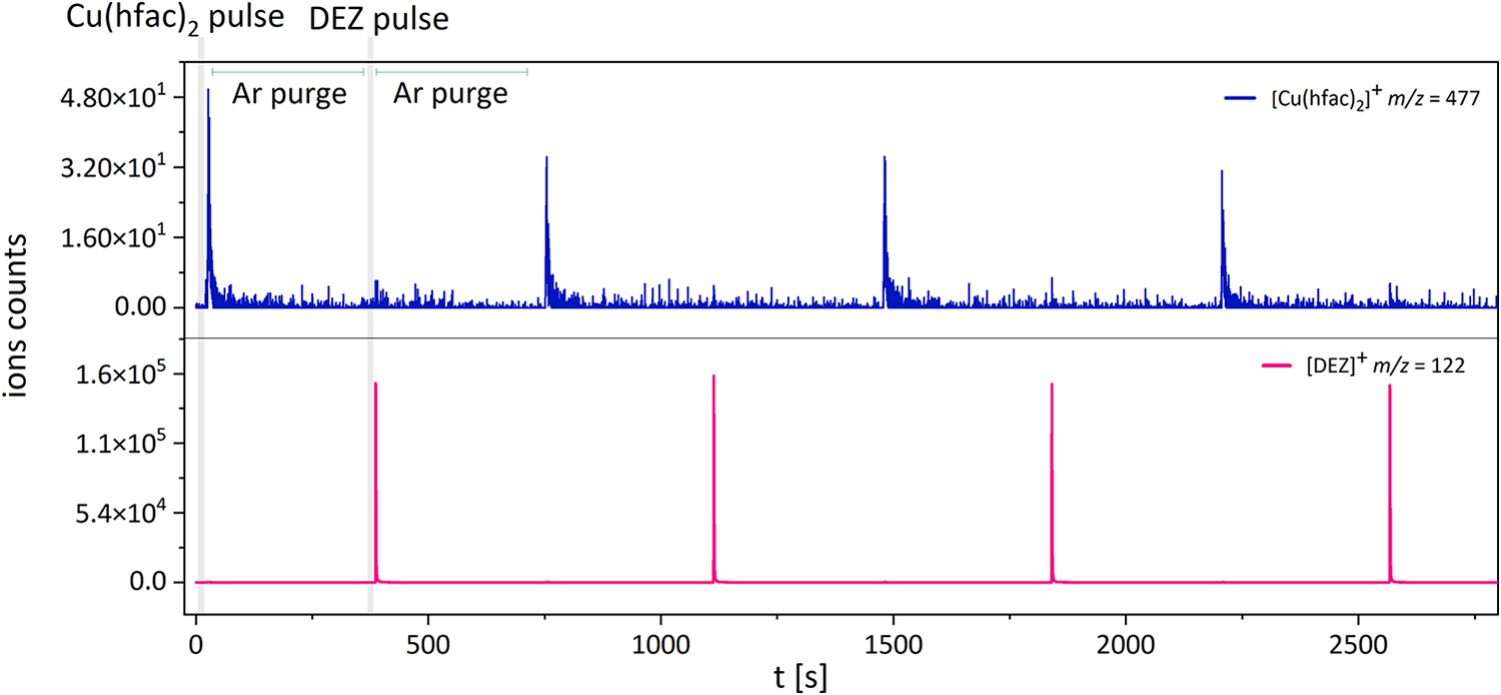
Temporal evolution (TS) of the parent ion signals of the reactants over four ALD cycles. Only the first ALD cycle is labeled in the graph.

The parent ions and fragments of the compounds listed in Table 1 were identified in the acquired mass spectra shown in Figure 6. The MS data for each half-cycle are divided into five distinct *m*/*z* regions, each displaying unique identifier peaks with the corresponding isotope patterns. The MS spectra revealed that both Zn-containing products, Zn­(hfac)_2_ and/or EtZn­(hfac), were formed during both half-cycles, as indicated by the most intense signal present and indicative of fluorine migration, [Zn­(hfac) – CF_2_]^+^ at *m*/*z* 221.

**Figure 6:**
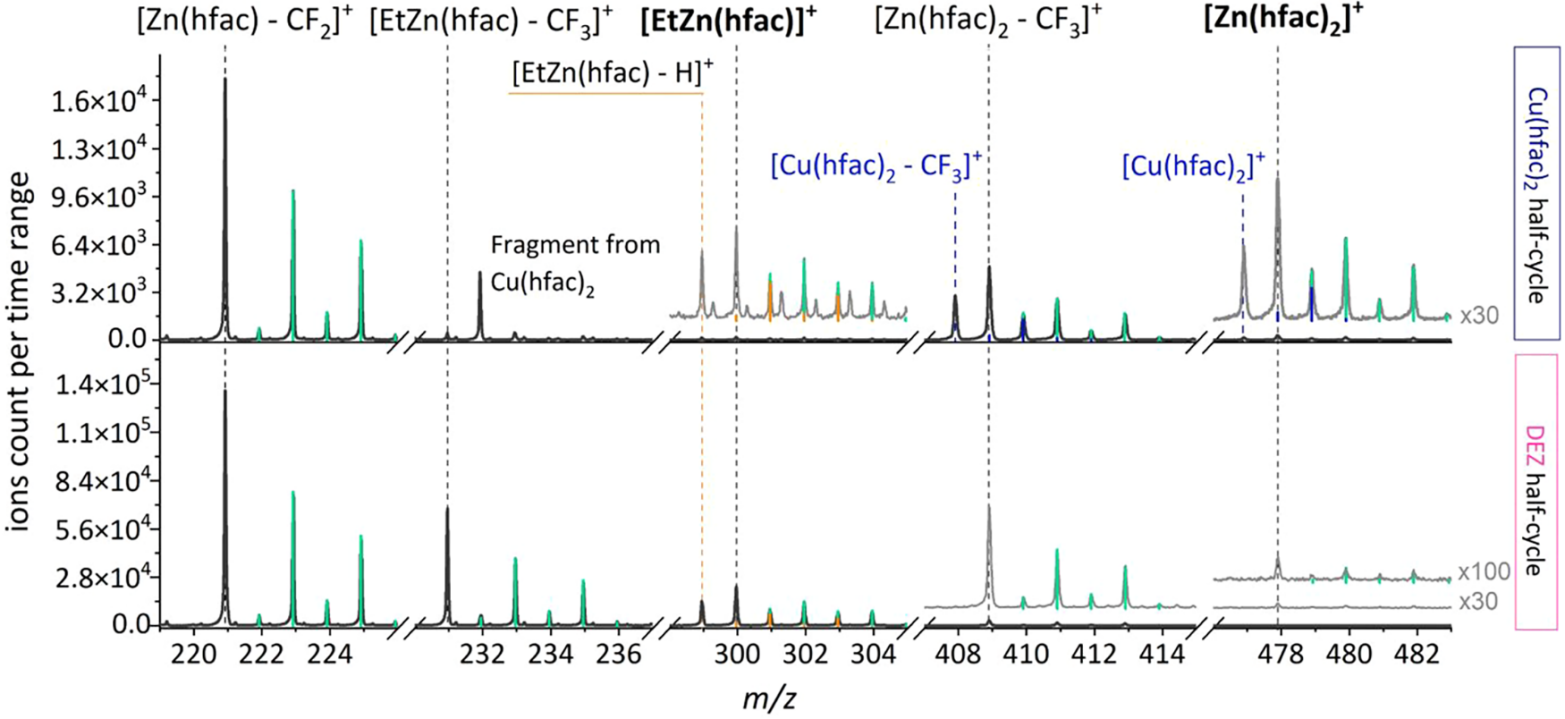
Relevant MS fragments and their isotopes from the Cu­(hfac)_2_ (top) and DEZ (bottom) ALD half-cycle. MS spectra are in dark gray, while light gray MS are enlargements. Parent ions of surface reaction volatile by-products Zn­(hfac)_2_ and EtZn­(hfac) are highlighted in bold. Green and orange lines represent isotope patterns of Zn, confirming the chemical identity of these species, likely excluding the occurrence of Cu-containing species in the DEZ half-cycle. Blue lines represent the isotope patterns of the Cu precursor. MS enlargement can be found in SI S2.5, Figure S6.

A detailed examination of the MS spectra collected during the Cu precursor half-cycle in Figure 6 revealed important insights into the by-product formation: (i) the MS signals indicate that both surface reaction volatile by-products, Zn­(hfac)_2_ and EtZn­(hfac), were formed during the Cu­(hfac)_2_ pulse. (ii) The parent ions of both species were detected with similar but weak intensities, highlighting significant fragmentation upon electron impact. The higher relative intensity of the peak at *m*/*z* 409, originating from the characteristic fragmentation of Zn­(hfac)_2_ indicates that Zn­(hfac)_2_ was formed in greater amounts than EtZn­(hfac). (iii) In contrast, the signal corresponding to the unique fragment of EtZn­(hfac) was notably very weak. Similarly, the MS data from the DEZ pulse (i) confirmed that both EtZn­(hfac) and Zn­(hfac)_2_ were generated as surface reaction volatile by-products during the reducing agent half-cycle. (ii) However, the more intense EtZn­(hfac) parent peak and the presence of its distinctive fragment at *m*/*z* 231 indicate that EtZn­(hfac) was the predominant product, while Zn­(hfac)_2_ was produced in much lower quantities.

To further confirm the presence of Zn species, isotopic pattern analysis was performed following the correction for overlapping isotope contributions, as highlighted in Figure 6. The observed isotopic distribution matched the expected natural abundance pattern for zinc-containing fragments, providing additional validation of the species identification; see SI S2.3 for a detailed explanation. Notably, the peak at *m*/*z* 299 required careful isotopic distribution analysis to determine the correct molecular assignment. Although [EtZn­(hfac) – H]^+^ and [EtCu­(hfac)]^+^ share the same nominal mass, isotopic pattern analysis, especially of the *m*/*z* 303 isotope, confirmed that the observed signal was originated from [EtZn­(hfac) – H]^+^. The copper isotopic pattern is primarily defined by two isotopes (^63^Cu and ^65^Cu), which do not contribute at +4, resulting in a negligible signal at *m*/*z* 303, thereby supporting the exclusion of Cu-containing species. Detailed analysis is provided in Supporting Information S2.6, Figure S7. The data presented suggests the formation of Zn-containing species rather than Cu-containing products in both half-cycles. Moreover, no fragment peaks that could be uniquely attributed to EtCu­(hfac) were detected, *i.e.*, [EtCu­(hfac) – CF_3_]^+^ at *m*/*z* 230, further confirming this conclusion. Additionally, a distinct fragment at *m*/*z* 232 was consistently observed during the Cu­(hfac)_2_ half-cycle. The isotopic signature of this compound (analysis in Supporting Information S2.7, Figures S8–S9) clearly rules out any contribution from metal-containing species, either Cu or Zn, but rather corresponds to a fluorinated organic species. Its presence during consecutive Cu­(hfac)_2_-only pulses confirms that it arises from fragmentation of the Cu precursor under electron ionization, rather than from surface reactions with DEZ. The exact structure of this fragment has been tentatively proposed based on its mass and the detection of related fragments at both higher and lower *m*/*z* values during consecutive Cu­(hfac)_2_-only pulses—none of which exhibit any Cu or Zn isotopic signatures.

Figure 7 shows the temporal evolution of the previously discussed signals over four ALD cycles. The periodic formation of Zn–containing by-products in each reactant pulse was clearly marked by the signal at *m*/*z* 221, corresponding to the [Zn­(hfac) – CF_2_]^+^ fragment in Figure 7. This fragment is originated by both the two by-products Zn­(hfac)_2_ and EtZn­(hfac) upon electron impact and may indicate that all hfac ligands are removed from the surface in the form of Zn­(hfac)­[X] species [X] = hfac or Et, resulting in Zn­(hfac)_2_ or EtZn­(hfac) compounds. Parent peak at *m*/*z* 478, attributed to Zn­(hfac)_2_, was very weak but appeared during both the copper precursor and DEZ pulse. The characteristic fragment [Zn­(hfac)_2_ – CF_3_]^+^ (*m*/*z* 409), which confirms the formation of Zn­(hfac)_2_, was also present in both pulses. The temporal evolution MS spectrum confirms again that Zn­(hfac)_2_ was formed in a bigger amount compared to EtZn­(hfac) during the Cu precursor half-cycle. In contrast, the detection of the intense parent peak of [EtZn­(hfac)]^+^ at (*m*/*z* 300) and its ionized fragment [EtZn­(hfac) – CF_3_]^+^ during exposure to DEZ clearly pointed to the formation of EtZn­(hfac) as the main by-product for the DEZ half-cycle.

**Figure 7:**
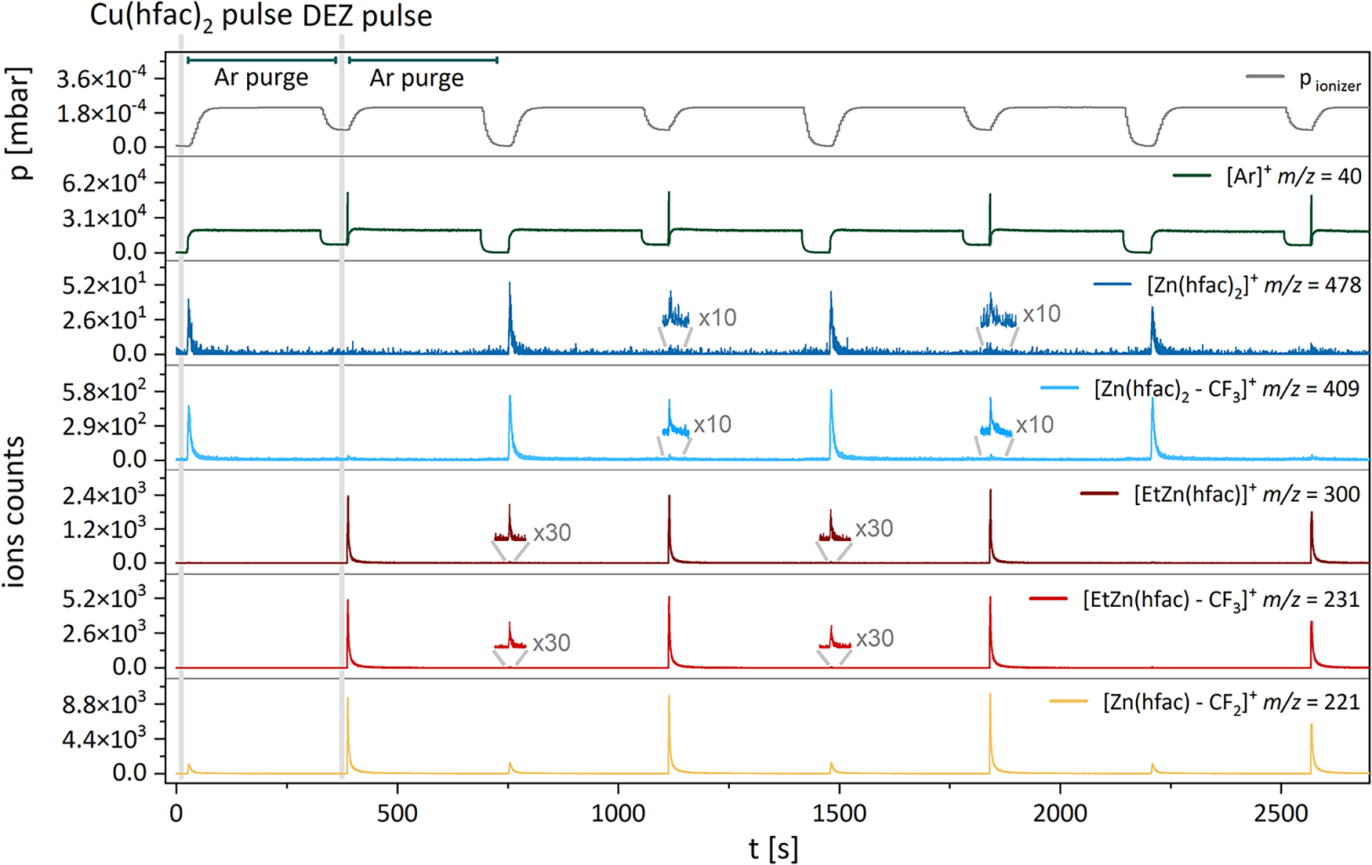
Temporal evolution of selected identifiers during the four ALD cycles. The graph is divided into several sections representing the different ALD subcycles: Cu­(hfac)_2_ pulse followed by Ar purge, and DEZ pulse followed by Ar purge. The identification of the peaks can be found in Table 1. Only the first ALD cycle is labeled in the graph.

### 3.4 Reaction Mechanism

We propose the Cu ALD growth model shown in Figure 8 based on the clear identification of released volatile surface reaction by-products by TOFMS and metallic copper as the main film constituent by XAS characterization. Figure 8 uses the notation introduced by Dey and Elliot^31^ in which each ALD half-cycle is conceptually divided into two steps (without imposing a strict sequence). In the first step, the incoming reactant molecules react with surface species remaining from the previous pulse, forming volatile by-products and metallic Cu. In the second step, once the surface species are exhausted, the excess reactant molecules adsorb onto the bare Cu surface (forming zinc-ethyl or hfac ligand adsorbed species). In reality, the first step in each half-cycles involves reactant molecules adsorption on the surface before ligand elimination step and, typically, for bulky ligands, dissociative chemisorption of the precursor, as in the second step.^32^ As a result, all of the surface reactions, namely redox reactions and ligand exchange, as well as surface processes such as adsorption, ligand diffusion, reordering, and desorption, see Figure 9, compete simultaneously with one another in each half-cycle and are ultimately governed by reaction kinetics. Nevertheless, it is legitimate to assume that each pulse is self-limiting and ends with the surface chemisorbed with an excess of the reactant. This formal two-step division is also employed in this study as a conceptual framework to explore all plausible reaction pathways, including the expected sequence of elementary steps, such as ligand exchange *versus* reductive elimination, that result in the formation of the observed volatile by-products.

**Figure 8:**
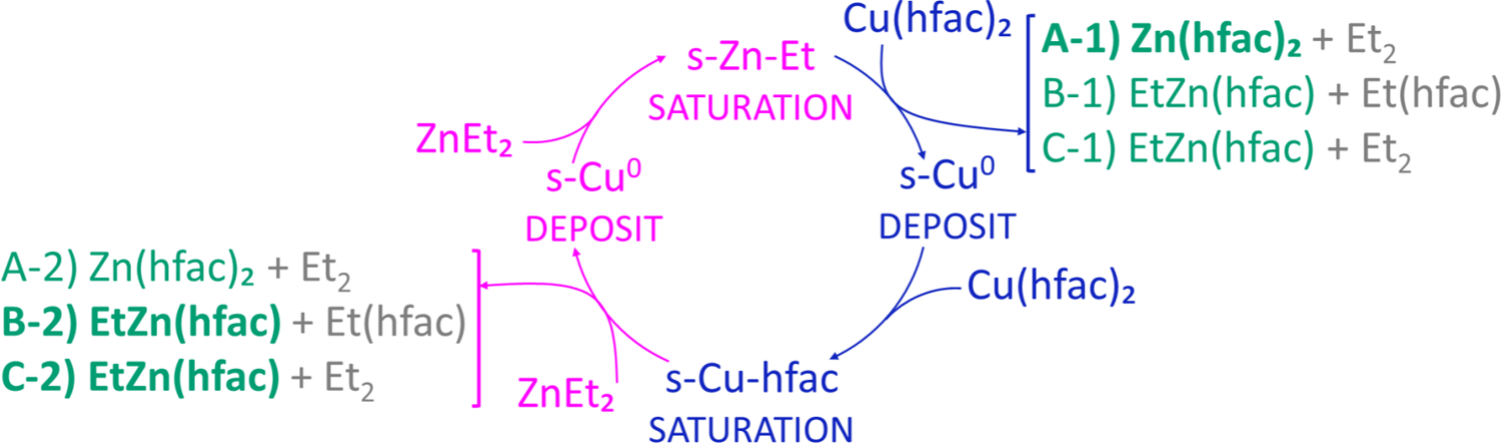
Schematic illustration of the Cu­(hfac)_2_ + ZnEt_2_ ALD cycle with surface reactions A, B, and C and detected volatile by-products as inferred from TOFMS measurements. Surface species are labeled with “s-”. During the Cu­(hfac)_2_ half-cycle, Zn­(hfac)_2_ was produced in higher amounts compared to the EtZn­(hfac) compound, while during the DEZ half-cycle, EtZn­(hfac) was the main volatile by-product generated. Color code: bold green: most intense TOFMS signal of molecular fingerprint; green: minor intense signal; gray: parent ion peak missing, but fragments visible.

**Figure 9:**
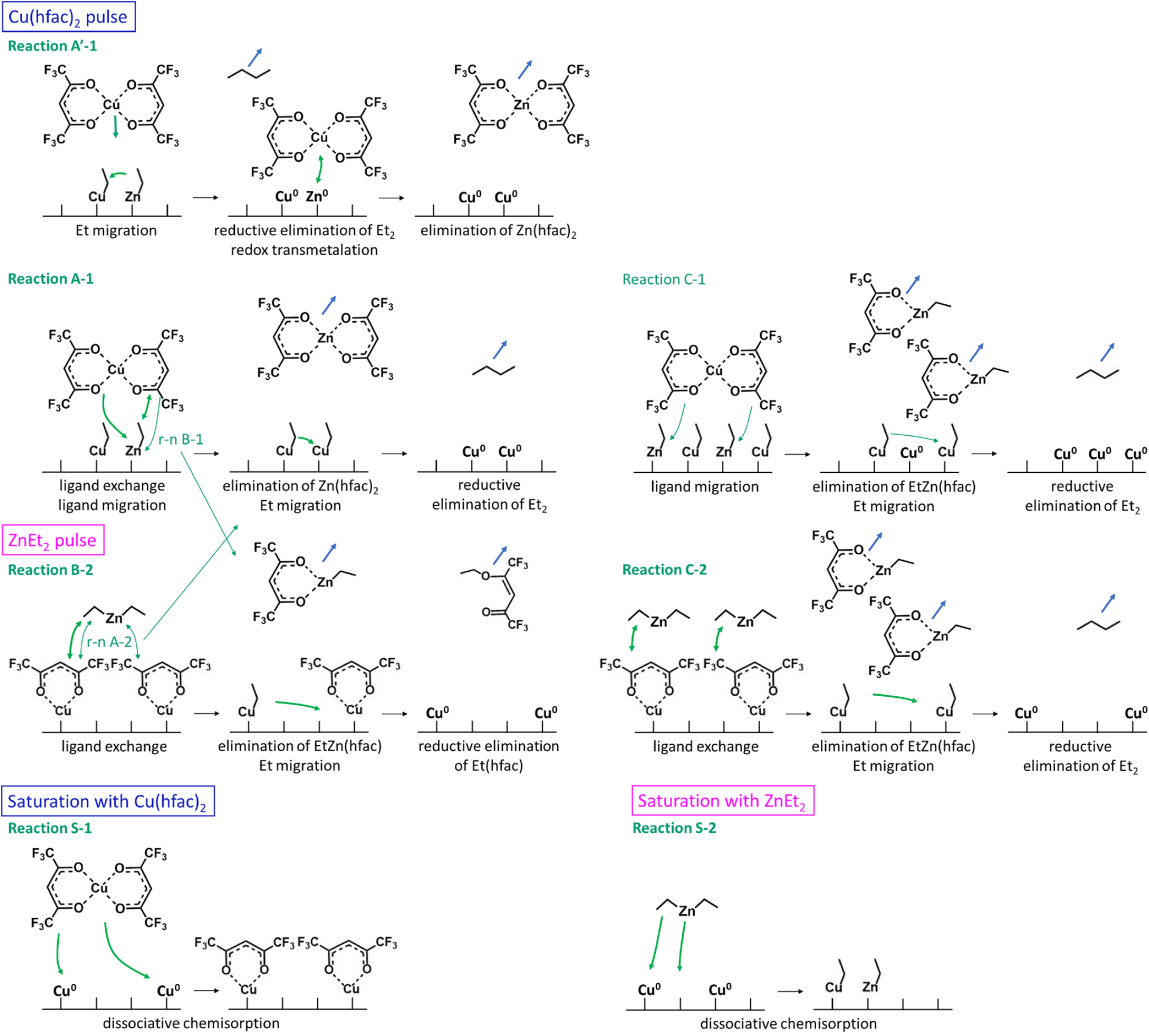
Schematic illustrations of possible surface reactions leading to the detected main volatile by-products: (A′-1) and (A-1) Zn­(hfac)_2_ + Et_2_ in Cu­(hfac)_2_ pulse; (B-2) EtZn­(hfac) + Et­(hfac) and (C-2) EtZn­(hfac) + Et_2_ in ZnEt_2_ pulse. Minor reaction pathways: (B-1), (C-1), and (A-2) in each pulse are indicated with light green. Each reaction pathway ends with the dissociative chemisorption of the reactant on the metallic Cu surface, resulting in the following adsorbed species: (S-1) s-Cu-hfac in a Cu­(hfac)_2_ pulse; (S-2) s-Zn-Et + s-Cu-Et in a ZnEt_2_ pulse.

The detection of Zn-containing by-products in the Cu precursor pulse confirms the presence of Zn-based species adsorbed onto the surface after the reducing agent pulse. Depending on the substrate, DEZ is known to dissociate at temperatures as low as 90 K to form adsorbed zinc metal and ethyl groups.^26–28,43^ At moderately low temperatures (100–150 °C) and in the absence of oxidizing species, Zn atoms remain on the Cu surface, forming a reactive Zn layer.^29^ DFT studies^30,32^ showed that DEZ molecules decompose into s-Cu-Et and s-Zn-Et fragments spontaneously upon adsorption onto Cu(111) without the presence of precursor fragments, as schematically shown in Figure 9, Reaction S-2. Following the formation of butane requires a rather high activation energy of +0.99 eV. On the other hand, another study^44^ demonstrated that butane elimination is the most energetically favorable pathway when excess DEZ is present on an intermetallic substrate, as proposed in reaction S’-2 in SI S4, Table S4. Therefore, at the process temperature of 190 °C, possible surface compounds at saturation with DEZ include s-Zn(0) and s-Zn-Et. However, further studies are needed to unambiguously determine the surface composition following each half-cycle. Nevertheless, both scenarios are considered plausible initial surface states for the Cu precursor reactions. The proposed reaction schemes are summarized in Supporting Information S4, Table S4.

During the Cu­(hfac)_2_ pulse, the precursor molecules react with the preadsorbed s-Zn(0) or s-Zn-Et surface species. As demonstrated by the MS data in Figures 6 and 7, the main volatile surface reaction by-product is Zn­(hfac)_2_. If only metallic Zn is present at the surface, a redox transmetalation reaction would occur, directly leading to Cu deposit, as schematically illustrated in step 2 of reaction A-1′ in Figure 9. In the presence of s-Zn-Et surface species, the mechanism is more complex and may proceed *via* two distinct pathways, following a DFT study based on a different Cu precursor, Cu­(dmap)_2_.^32^ In reaction A′-1, exposure to Cu precursor molecules induces migration of s-Et groups, forming butane, followed by redox transmetalation. In reaction A-1, the s-Et groups are involved in the transmetalation reaction *via* ligand exchange with the Cu precursor, followed by Zn­(hfac)_2_ desorption and reductive elimination of butane. If only partial ligand exchange mechanism takes place, the heteroleptic volatile by-products EtZn­(hfac) or EtCu­(hfac) could be produced, followed by the reductive elimination that leads to desorption of Et­(hfac) molecules (reactions B-1 in Figure 9 and D-1 in SI S4, Table S4) or, in the presence of high concentrations of s-Et groups, to butane (reaction C-1 in Figure 9 and E-1 in SI S4, Table S4). According to the reaction stoichiometry (SI S4, Table S4), the formation of EtCu­(hfac) would result in Zn(0) deposition. However, this reaction pathway was ruled out as TOFMS detected no isotopic fingerprint of EtCu­(hfac) during this pulse, and XAS analysis confirmed only trace amounts of Zn in the deposited thin film.

In the Cu­(hfac)_2_ precursor half-cycle, steric hindrance from the bulky ligands, possibly combined with the surface densely covered with s-Et groups, may limit the efficiency of both full and partial ligand exchange. Therefore, the most probable reaction pathway is reaction A′-1, in which s-Et groups are released before transmetalation, allowing the Cu precursor to interact freely with s-Zn(0). DFT calculations have shown that butane formation from a high concentration of s-Et groups or from crowded s-ZnEt_2_, facilitated by ligand cooperation, is highly exothermic.^30,32^ An additional argument favoring reaction A′-1 over A-1 is that the competing pathways B-1 and C-1 play only a minor role, in contrast to the DEZ pulse, where the primary by-product is heteroleptic EtZn­(hfac). The detection of [EtZn­(hfac)]^+^ ions in this half-cycle confirms that partial ligand exchange does occur but remains a secondary pathway. The A′-1 reaction scenario could be followed by the half-ligand exchange, which appears easier than full ligand exchange, with the sparse s-Zn-Et groups remaining after the majority have been eliminated as butane.

At the end of the Cu­(hfac)_2_ pulse, the surface is composed of s-Cu-hfac species, as evidenced by the detection of hfac-containing by-products in the subsequent pulse (Figures 6 and 7). As previously indicated by DFT calculations,^32,45^ Cu­(hfac)_2_ reacts with the underlying Cu(0), possibly *via* dissociative chemisorption, as schematically illustrated in Figure 9 and Reaction S-1. DFT results show that this reaction is feasible at the process temperature, with an activation energy of +0.44 eV for Cu­(dmap)_2_ on the Cu(111) surface,^32^ and +0.59 eV for Cu­(acac)_2_ on the Cu(110) surface.^46^ While the dissociation of the second ligand is endothermic for Cu­(dmap)_2_, it is exothermic for Cu­(acac)_2_, a precursor with a chemical structure similar to the one used in this study, with an energy barrier of +0.56 eV. This indicates that Cu­(acac)_2_ undergoes sequential dissociation and reduction on the Cu(110) surface: s-Cu­(acac)_2_ → s-Cu­(acac) + s-(acac) → s-Cu(0) + 2 s-(acac). Further studies dedicated specifically to Cu­(hfac)_2_ are needed to determine the extent of ligand dissociation at this stage.

In the DEZ half-cycle, the partial ligand exchange pathway, leading to the formation of EtZn­(hfac) (reaction B-2 or C-2, Figure 8), becomes the dominant mechanism. This shift occurs because the incoming ZnEt_2_ molecules, being smaller than the Cu­(hfac)_2_ precursor molecules and highly reactive, interact more readily with the surface already functionalized with hfac ligands. As a result, steric hindrance is no longer a limiting factor, making partial ligand exchange highly efficient. There are two possible routes leading to the formation of this heteroleptic by-product: reaction B-2, with elimination of Et­(hfac), or reaction C-2, marking a higher concentration of DEZ with elimination of butane. A clear distinction between these pathways could not be defined, as the parent peaks of the additional by-products could not be unambiguously assigned. The formation of EtCu­(hfac), and consequently mechanisms D-2 and E-2 (SI S4, Table S4), were ruled out.

As summarized in Figure 9, redox transmetalation (reaction A-1′) or full ligand exchange transmetalation (reaction A-1) dominates during the Cu precursor pulse, producing Zn­(hfac)_2_ and butane as volatile products. Under the assumption that the surface is terminated with Et groups following the DEZ pulse, the half-reaction of the Cu precursor pulse aligns with the hypothetical Reaction 1.1

$$s‐\mathrm{Zn}‐\mathrm{Et}+s‐\mathrm{Cu}‐\mathrm{Et}+2\mathrm{Cu}{\left(\mathrm{hfac}\right)}_{2\left(g\right)}\to 2s‐\mathrm{Cu}‐\mathrm{hfac}+s‐\mathrm{Cu}\left(0\right)+\mathrm{Zn}{\left(\mathrm{hfac}\right)}_{2\left(g\right)}+{\mathrm{Et}}_{2\left(g\right)}$$

In contrast, the introduction of DEZ triggers a partial ligand exchange mechanism, leading to the formation of Et­(hfac) in reaction B-2 or butane in reaction C-2. Two alternative half-reactions are thus proposed, with the first matching Reaction 2.2

$$2s‐\mathrm{Cu}‐\mathrm{hfac}+2{\mathrm{ZnEt}}_{2\left(g\right)}\to s‐\mathrm{Zn}‐\mathrm{Et}+s‐\mathrm{Cu}‐\mathrm{Et}+s‐\mathrm{Cu}\left(0\right)+\mathrm{EtZn}{\left(\mathrm{hfac}\right)}_{\left(g\right)}+\mathrm{Et}{\left(\mathrm{hfac}\right)}_{\left(g\right)}$$

$$2s‐\mathrm{Cu}‐\mathrm{hfac}+3{\mathrm{ZnEt}}_{2\left(g\right)}\to s‐\mathrm{Zn}‐\mathrm{Et}+s‐\mathrm{Cu}‐\mathrm{Et}+s‐\mathrm{Cu}\left(0\right)+2\mathrm{EtZn}{\left(\mathrm{hfac}\right)}_{\left(g\right)}+{\mathrm{Et}}_{2\left(g\right)}$$

Thus, throughout the Cu ALD cycle using Cu­(hfac)_2_ and DEZ, at least two distinct mechanisms operate, each depending on the surface composition in each half-cycle. The full range of by-products formed in this process remains unclear. Therefore, there are two possible overall reactions for this process

3 $$\mathrm{Cu}{\left(\mathrm{hfac}\right)}_{2\left(g\right)}+{\mathrm{ZnEt}}_{2\left(g\right)}\to s‐\mathrm{Cu}\left(0\right)+\frac{1}{2}\mathrm{Zn}{\left(\mathrm{hfac}\right)}_{2\left(g\right)}+\frac{1}{2}\mathrm{EtZn}{\left(\mathrm{hfac}\right)}_{\left(g\right)}+\frac{1}{2}\mathrm{Et}{\left(\mathrm{hfac}\right)}_{\left(g\right)}+\frac{1}{2}{\mathrm{Et}}_{2\left(g\right)}$$

4 $$\mathrm{Cu}{\left(\mathrm{hfac}\right)}_{2\left(g\right)}+\frac{3}{2}{\mathrm{ZnEt}}_{2\left(g\right)}\to s‐\mathrm{Cu}\left(0\right)+\frac{1}{2}\mathrm{Zn}{\left(\mathrm{hfac}\right)}_{2\left(g\right)}+\mathrm{EtZn}{\left(\mathrm{hfac}\right)}_{\left(g\right)}+{\mathrm{Et}}_{2\left(g\right)}$$

Of note is that the relative intensities of the by-products in each half-cycle (Figure 7) suggest that, under our experimental conditions, most surface reaction by-products are released during the DEZ pulse, while the Cu precursor pulse generates fewer. This imbalance could indicate a mechanism resembling an abbreviated ALD cycle in which the DEZ reaction might terminate after copper reduction, making this step the main contributor to film growth. Such an interpretation could also explain the lower GPC observed in our study. However, further analysis and a quantitative assessment of by-product formation are required to support this hypothesis.

## 4 Conclusions

The reaction mechanism of the Cu ALD process from Cu­(hfac)_2_ and ZnEt_2_ was investigated *via* *in situ* TOFMS and was supported by XAS analysis of the deposited film. The surface reaction volatile by-products of each half-cycle, along with the chemical composition of the resulting film, were identified, providing key insights into the process mechanism.

TOFMS analysis, supported by molecular parent peaks identification and isotope pattern fitting, provided a robust framework for distinguishing reactants and volatile by-products amid significant mass interferences. The identification of a unique fluorine migration fragmentation pathway during ionization, present in Zn-containing species but absent in Cu­(hfac)_2_, enabled the selection of distinct *m*/*z* markers. Zn­(hfac)_2_ was identified as the dominant volatile by-product during the Cu precursor pulse, while EtZn­(hfac) was predominant during the DEZ pulse. However, both by-products were detected in each half-cycle. The absence of an isotope pattern corresponding to EtCu­(hfac) in both pulses ruled out its formation. Additional organic by-products butane or Et­(hfac) could not be clearly identified due to the absence of the parent ion peaks, although the ionization fragments, but not unique identifiers, were visible.

XAS analysis confirmed that the deposited films consisted predominantly of metallic Cu, with Zn contamination ≤ 1 at %, indicating effective Zn removal in each cycle. A ∼ 4 nm Cu oxide layer was detected, attributed to air exposure during sample transfer rather than to the ALD process itself. Total fluorescence yield analysis showed negligible carbon and fluorine incorporation within the film, confirming the efficient removal of Et and hfac groups in each half-cycle. In contrast, the total electron yield spectra showed a noticeable presence of carbon and some fluorine residues on the film surface, likely originating from contamination due to air exposure and from residual Cu­(hfac)_2_ or hfac ligand adsorbed molecules.

The resulting growth model confirms the saturation surface composition: hfac ligands remain on the surface after Cu precursor exposure, while Zn species (metallic Zn or Zn-Et groups) persist at the end of the DEZ pulse. Each half-cycle follows a different reaction mechanism, underscoring the role of surface composition. The Cu precursor pulse appears to be governed by redox transmetalation or full ligand exchange transmetalation, leading to Zn­(hfac)_2_ as the primary volatile by-product. However, the transmetalation in this step is limited to the amount of Zn species present and gives a minor contribution to the Cu deposit. In contrast, DEZ exposure appears to trigger a partial ligand exchange mechanism, producing EtZn­(hfac) as the main channel for the metallic Cu deposition.

By correlating *in situ* TOFMS ALD process monitoring with XAS analysis of the resulting film, this work provides insights into the surface-dependent chemistry of each half-cycle. These findings contribute to a deeper understanding of the ALD growth process of metals and offer valuable guidance for future process control and optimization. In the future, we want to extend to quantitative *in situ* TOFMS studies, including the early stage phase of Cu ALD on substrate surfaces.

## Supplementary Material



## Notes

Experimental conditions of the Cu ALD process during TOFMS; TOFMS and XAS data treatment and analysis; control ALD process; list of possible reaction mechanisms between Cu­(hfac)_2_ and DEZ molecules (PDF)
