# Supplementary material for: Reaction Mechanisms in Copper Atomic Layer Deposition Using Copper(II) Hexafluoroacetylacetonate and Diethylzinc via In Situ Time-of-Flight Mass Spectrometry
Source: Chem Mater. 2025 Sep 11;37(18):7264–76. doi: 10.1021/acs.chemmater.5c01521 (PMC12461784; doi:10.1021/acs.chemmater.5c01521)
Supplement: Supplementary file 1 [file cm5c01521_si_001.pdf]

SUPPORTING INFORMATION

"Reaction Mechanisms in Copper Atomic Layer Deposition using Copper(II) Hexafluoroacetylacetonate and Diethylzinc via *in situ* Time-of-flight Mass Spectrometry"

Camilla Minzoni[1,2, ‡], Sylwia Klejna[3, ‡], Krzysztof Mackosz[1], Caroline Hain[1], Aleksandra Figura-Jagoda[3], Aleksandra Szkudlarek[3], Marcin Sikora[4], Andreas Werbrouck[5], Ramakrishna Ramisetty[6], Patrik Hoffmann[1,2], Ivo Utke[1]

[1] Empa, Swiss Federal Laboratories for Materials Science and Technology, Feuerwerkerstrasse 39, 3602 Thun, Switzerland

[2] EPFL, École Polytechnique Fédérale de Lausanne, Station 17, 1015 Ecublens, Switzerland

[3] AGH University of Krakow, Academic Centre for Materials and Nanotechnology, Al. Mickiewicza 30, 30-059 Krakow, Poland

[4] National Synchrotron Radiation Centre SOLARIS, Jagiellonian University, Czerwone Maki 98, 30-392 Krakow, Poland

[5] Materials Science and Engineering Institute, University of Missouri, Laferre Hall, 416 S 6th St, Columbia, MO 65201, USA

[6] TOFWERK AG, Schorenstrasse 39, 3645 Thun, Switzerland

‡ Shared first authorship

TABLE of CONTENTS

- **S1 Experimental Conditions of the ALD Process during TOFMS S4-S6**
  - S1.1 Detailed ALD cycle and process parameter S4-S5
  - S1.2 Dehydration process S6
- **S2 TOFMS Data Analysis S7-S17**
  - S2.1 Calibration procedure S7-S8
  - S2.2 MS data averaging procedure S9-S10
  - S2.3 TOFMS mass resolution and isotope profiling. S10-S11
  - S2.4 Control ALD experiment for pressure impact on the baseline TOFMS signal S11-S12
  - S2.5 Enlargement of MS during the two half-cycles S13
  - S2.6 Fragment peak at *m/z* = 299 S14
  - S2.7 Fragment peak at *m/z* = 232 S15-S17
- **S3 XAS Data Analysis S18-S19**
- **S4 Reaction Mechanism S20**

S1 Experimental Conditions of the ALD Process during TOFMS

S1.1. Detailed ALD cycle and process parameter

Table S1. Detailed ALD cycle and process parameters used during TOFMS measurements. The symbol "-" stands for an action.

	ALD cycle			
		Setting	Ar flow / sccm	Time
$Cu(hfac)_2$ half-cycle	1.	Ar set point	0	-
		Waiting time	0	1 min
	2.	Exhaust valve close	0	-
		Waiting time	0	500 ms
	3.	$Cu(hfac)_2$ pulse duration	0	300 ms
		Exposure time	0	1 s
	4.	Ar set point	15	-
		Waiting (exposure) time	15	1 s
	5.	Exhaust valve open	15	-
		Waiting time	15	1 s
	6.	Ar set point	100	-
	7.	Purge	100	5 min
DEZ half-cycle	1.	Ar set point	30	-
		Waiting time	30	1 min
	2.	Exhaust valve close	30	-
		Waiting time	30	100 ms
	3.	DEZ pulse duration	30	50 ms
		Exposure time	30	2 s
	4.	Exhaust valve open	30	-
		Waiting time	30	1 s
	5.	Ar set point	100	-
	6.	Purge	100	5 min

ALD process parameters	
Chamber pressure	Measured
p background (0 sccm)	2.3×10^{-2} mbar
p (purge 100 sccm Ar)	2.9×10^{-1} mbar
p (beginning step 3 $Cu(hfac)_2$)	2.3×10^{-2} mbar
p (before step 5 $Cu(hfac)_2$)	6.3×10^{-2} mbar
p (beginning step 3 DEZ)	1.3×10^{-1} mbar
p (before step 4 DEZ)	5.9×10^{-1} mbar
**Temperature**	**Setting**
Temperature $Cu(hfac)_2$	80°C
Temperature DEZ	5°C
Temperature Ar lines	100°C
Temperature substrate	190°C
Temperature walls	120°C
Temperature exhaust	120°C
Temperature bellow	100°C

Figure S1 shows the process scheme for one full ALD cycle during the TOFMS measurement, detailing the sequence of both reactants' pulses along with the timing of exhaust valve closure and opening. Moreover, the MS signal over time is shown for both reactants (*m/z* 477 for $[Cu(hfac)_2]^+$ and 122 for $[DEZ]^+$) and for Ar (*m/z* 40), highlighting the changes of the Ar signal due to the exhaust valve closure and opening.

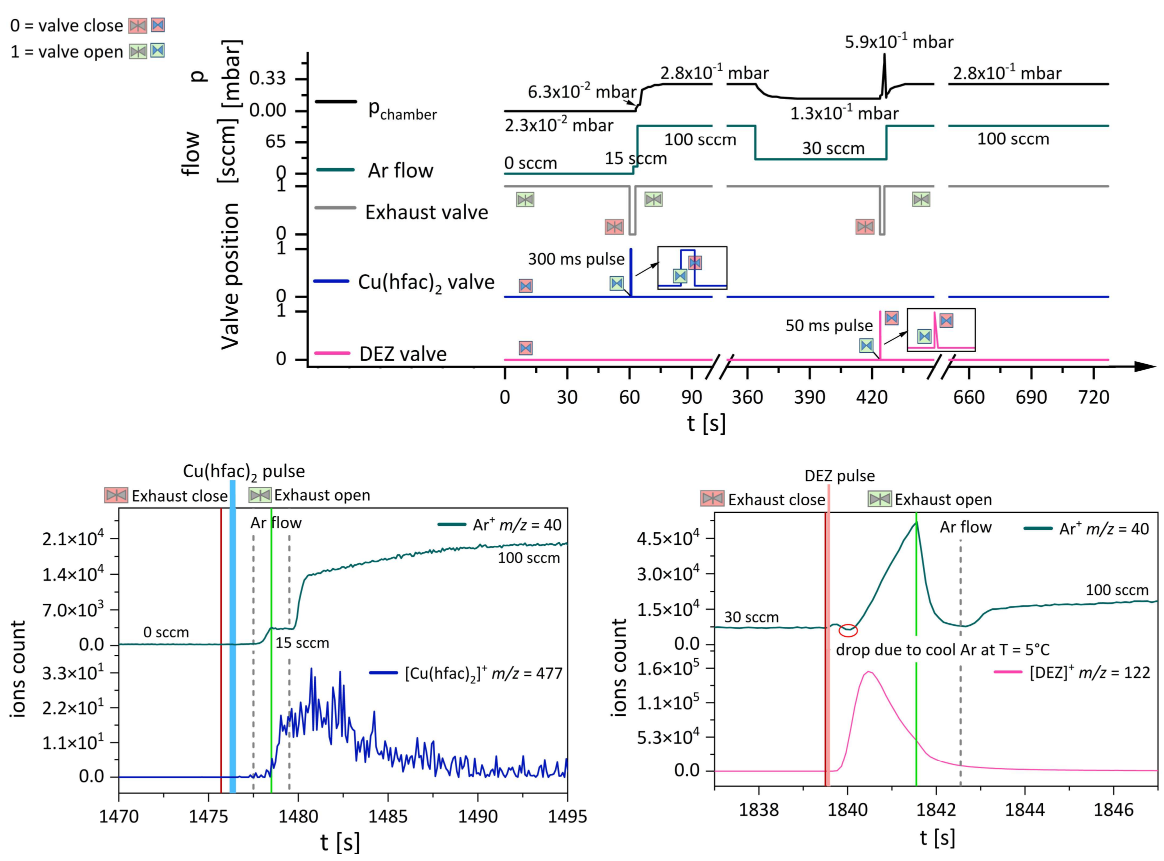

Figure S1. (top) ALD time-step cycle plot with each reactant pulse, exposure, and Ar flow over time. (bottom) MS time evolution of the Ar peak along with the $Cu(hfac)_2$ parent peak during the $Cu(hfac)_2$ half-cycle (left) and with DEZ parent peak (right) during the Et_2Zn half-cycle. The respective pulsing, exposure, purge sequences are indicated.

S1.2. Dehydration process

The dehydration process was carried out using a vacuum system ($p_{background}$ = 5×10^{-5} mbar), in which the ALD reservoir containing the $Cu(hfac)_2$ compound (solid powder) were connected to pump the water out. A grid was positioned at the outlet of the reservoir to prevent powder dispersion into the vacuum system. The reservoir was cooled in a water bath, and magnetic stirring was also employed to ensure complete dehydration of the entire powder. The dehydration process was monitored by observing the color change of the $Cu(hfac)_2$ compound, which shifts from an intense green in its hydrated form to blue upon dehydration.

S2 TOFMS Data Analysis

S2.1. Calibration procedure

Mass calibration was performed to ensure accuracy across the monitored mass range (*m/z* = 15 – 500). The TOFMS system was calibrated using a five-point calibration method. In the low-mass region, three reference points were selected: Zn^{+} parent peak *m/z* 64 and two background signals from the carrier gas, specifically Ar^{+} *m/z* 40 and Ar^{++} *m/z* 20. In the higher mass range, two characteristic fragments from the $Cu(hfac)_2$ were used, $[Cu(hfac) – CF_3]^{+}$ at *m/z* 201 and $[Cu(hfac)_2 – CF_3]^{+}$ at *m/z* 408. Peak fitting analysis was performed using a Pseudo-Voigt function. The fitted peaks showed excellent agreement with the experimental data, resulting in relative mass errors below 10 ppm across all calibrant peaks. This high level of accuracy demonstrates the reliability and precision of the mass calibration across the entire monitored mass range, ensuring confident mass assignments for unknown species. The fitting procedure for each calibrant peak required an initial time-of-flight (TOF) position estimate. This initial position t_0 was calculated using preliminary calibration coefficients and the known exact mass of the calibrant species. To optimize the peak fit, this initial position was then refined by identifying the maximum signal intensity within a defined search window of $t_0 \pm t_{psr}$, where t_{psr} represents the peak search range (illustrated by blue shading in the Figure S2). The identified maximum signal position was then used as the refined starting point for the subsequent peak fitting procedure. The specified exact masses and fitted TOF positions for the calibrant peaks were subsequently used to perform the fit to the calibration function. The results of the calibration fit are shown in Figure S2.

a)

Calibrants	m/Q defined	Initial TOF position	Fitted TOF position	1e6*(mExact-mFit)mExact (ppm)
Ar^{++}	19.980643	8380.92	8380.9	10
Ar^{+}	39.961835	11653.8	11654	3
Zn^{+}	63.928593	14612.9	14613	5
$[C_4HCuF_3O_2]^+$	200.922461	25536.5	25536	5
$[C_9H_2CuF_9O_4]^+$	407.910535	36180.5	36180	3

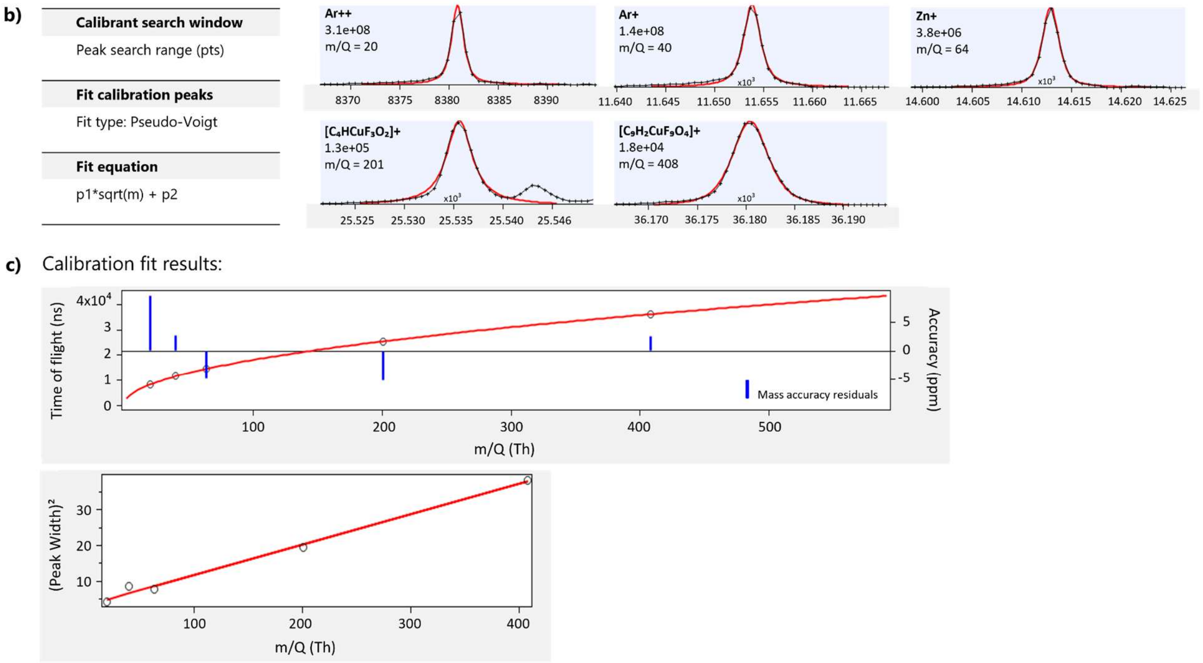

Figure S2. (a) Calibration table showing theoretical *m/z* values (*m/z* defined), initial and fitted TOF peak position and relative error in ppm. (b) MS peak fitting for the five calibrant species. The experimental data (black dots) are fitted with Pseudo-Voigt functions (red lines). The calibration parameters are shown on the left panel. (c) Residual analysis graph (top): the red line describes the relationship between TOF and mass-to-charge ratio while the blue sticks describe the deviation, in mass space, of the given exact masses of the calibrant peaks from the values given by the fit coefficients. Mass calibration curve (bottom), showing a linear trend between the square of the fitted peak width and *m/z*.

S2.2 MS data averaging procedure

One mass spectrum was collected every 100 ms, each generated by averaging 2290 waveforms, with one waveform recorded during each TOF extraction. The mass spectra (MS) of the two half-cycles were generated by averaging the time-series (TS) signal over the pulse duration of each reagent. For both $Cu(hfac)_2$ and DEZ, the time window was defined based on the start and end points of their respective peaks; these time boundaries were established using the following threshold:

$$\text{Threshold} = 2 \times \overline{\text{background}} + 10 \times S_{\text{background}} \tag{S1}$$

$\overline{\text{background}}$ = mean of the background $S_{\text{background}}$ = background standard deviation

Table S2. Time window used for averaging the MS during $Cu(hfac)_2$ and DEZ half-cycle.

	$Cu(hfac)_2$ half-cycle	$ZnEt_2$ half-cycle
**Start time** / s	1478.13	1839.56
**End time** / s	1488.93	1883.46

Figure S3 highlights the time window used for generating the mass spectra during the $Cu(hfac)_2$ and DEZ pulses.

a)

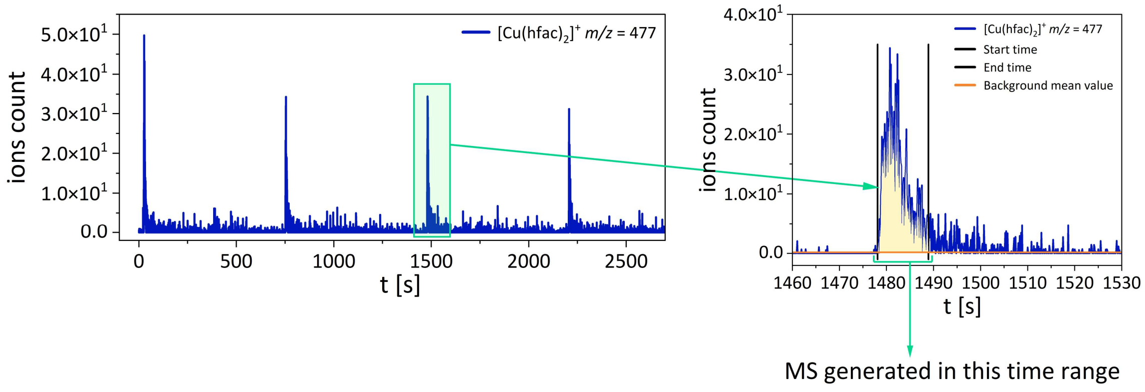

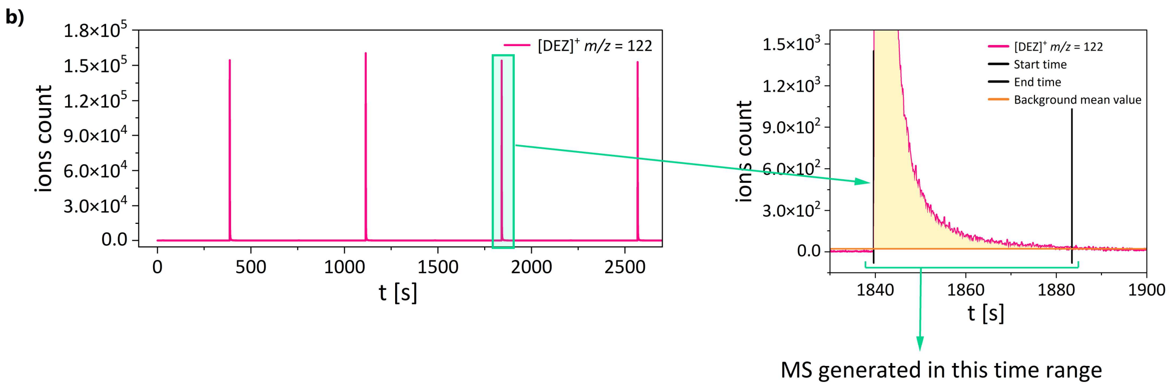

Figure S3. Time window used for generating the correlated mass spectra during the $Cu(hfac)_2$ pulse (a) and the DEZ pulse (b).

S2.3 TOFMS: mass resolution and isotope profiling

The mass difference between $Cu(hfac)_2$ (m = 476.90 u) and $Zn(hfac)_2$ (m = 477.90 u) is only $\Delta m = 9.996\times10^{-1}$, requiring a mass resolving power of approximately $R = m/\Delta m = 477$ for peak differentiation. The required resolution is well within the TOFMS system's specification of 4000, allowing for precise separation of the two parent peaks, as demonstrated in Figure S4.

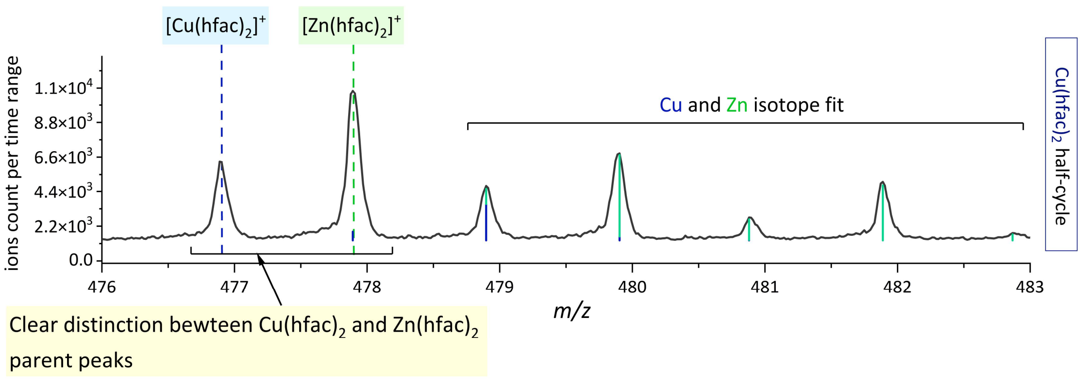

Figure S4. High mass resolution of TOFMS detector allows for the discrimination between Cu and Zn complexes. Here it is shown the distinction between $Cu(hfac)_2$ and $Zn(hfac)_2$ molecular peaks.

The co-occurrence of Cu and Zn analogous compounds results in spectral overlap due to the presence of isotopes which generate additional peaks. Detailed isotope profiling is therefore required, and it was based on the natural abundance of each element in the observed fragment. To deconvolute overlapping peaks, the isotopic contribution of Cu-containing fragment at each mass-to-charge ratio was calculated based on its natural isotopic distribution and then subtracted from the total peak intensity to obtain the true intensity of the corresponding Zn-containing peak. Table S3 gives an example of isotope contributions for the $Cu(hfac)_2$ and $Zn(hfac)_2$ masses.

Table S3. Isotope contribution for $Cu(hfac)_2$ and $Zn(hfac)_2$ masses.

***m/z* peak**	**$Cu(hfac)_2$**	**$Zn(hfac)_2$**
477	100%	-
478	11%	11%
479	46%	58%
480	5%	14%
481	0.6%	39%
482	0.04%	1.7%
483	-	0.17%

S2.4 Control ALD experiment for pressure impact on the baseline TOFMS signal

A control experiment was performed to assess the potential impact of pressure-related fluctuations in the signal. This experiment simulated an ALD process without the actual pulsing of the reactants (closed manual valves of the reactant reservoirs) and was already performed by Hornsveld et al.[1] to test the pressure dependence of the baseline mass signal in their quadrupole mass spectrometer. In our case, the Ar peak intensity and ionizer pressure were continuously monitored as indicators, while also tracking parent peaks of the two reagents and of the expected surface reaction volatile by-products to verify signal stability throughout the pseudo-ALD cycles.

Figure S5 reveals that butane (C_4H_{10}) was the only molecule exhibiting pressure-dependent variations in its baseline value, while all other monitored species maintained consistent baseline signal levels throughout the experiment.

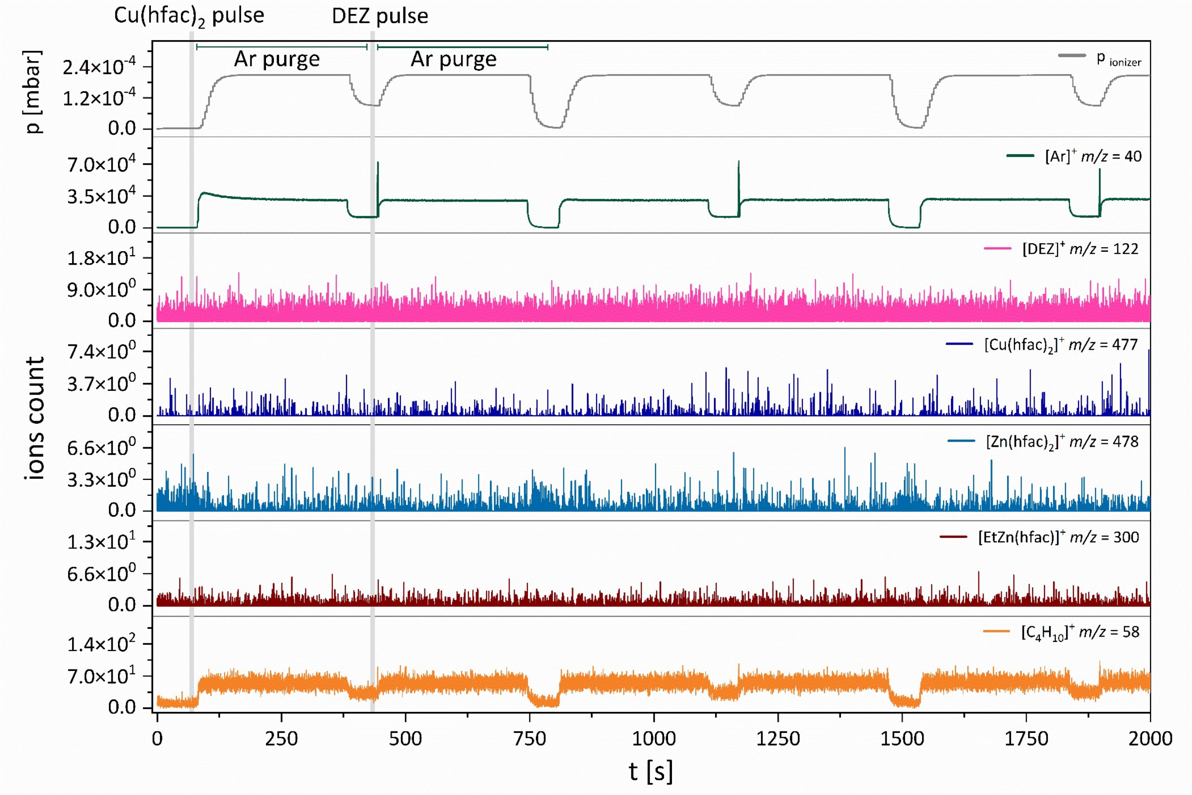

Figure S5. Temporal evolution of pressure changes in the mass spectrometer ionizer (top) and of the base lines of parent peaks of the two reagents and of the expected surface reaction volatile by-products during three ALD cycles. The graph is divided into several sections representing the different ALD sub-cycles: Cu(hfac)$_2$ pulse followed by Ar purge, DEZ pulse followed by Ar purge. The first two Ar purge cycles are indicated.

S2.5 Enlargement of MS during the two half-cycles

In Figure S6 the enlargement of the mass spectra during the two half-cycles are reported.

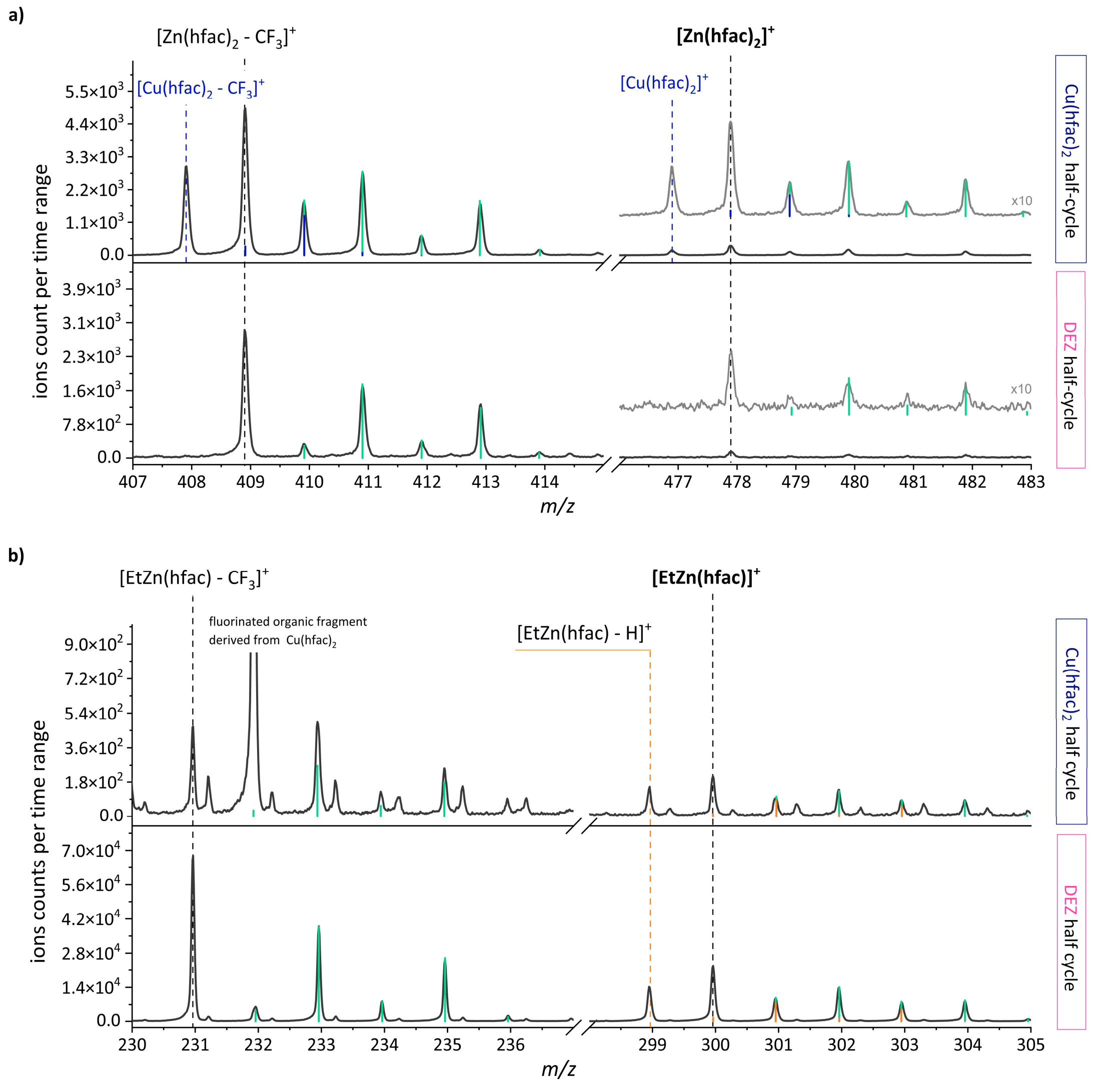

Figure S6. MS data from *m/z* = 406 – 483 (a) and from *m/z* = 230 – 305 (b) from Cu(hfac)$_2$ (top) and DEZ (bottom) ALD half-cycle. Parent ions of surface reaction volatile by-products Zn(hfac)$_2$ and EtZn(hfac) are highlighted in bold. Green and orange lines represent isotope patterns of Zn, confirming the chemical identity of these species, likely excluding the occurrence of Cu-containing species in the DEZ half-cycle. Blue lines represent isotope patterns of the Cu precursor.

S2.6 Fragment peak at *m/z* = 299

The fragment peak at m/z 299 observed in the mass spectrum required careful isotopic distribution analysis to determine the correct molecular assignment: this isotopic pattern analysis, especially of the *m/z* 303 isotope, confirmed that the observed signal was originated from [EtZn(hfac) – H]$^+$, as shown in Figure S7.

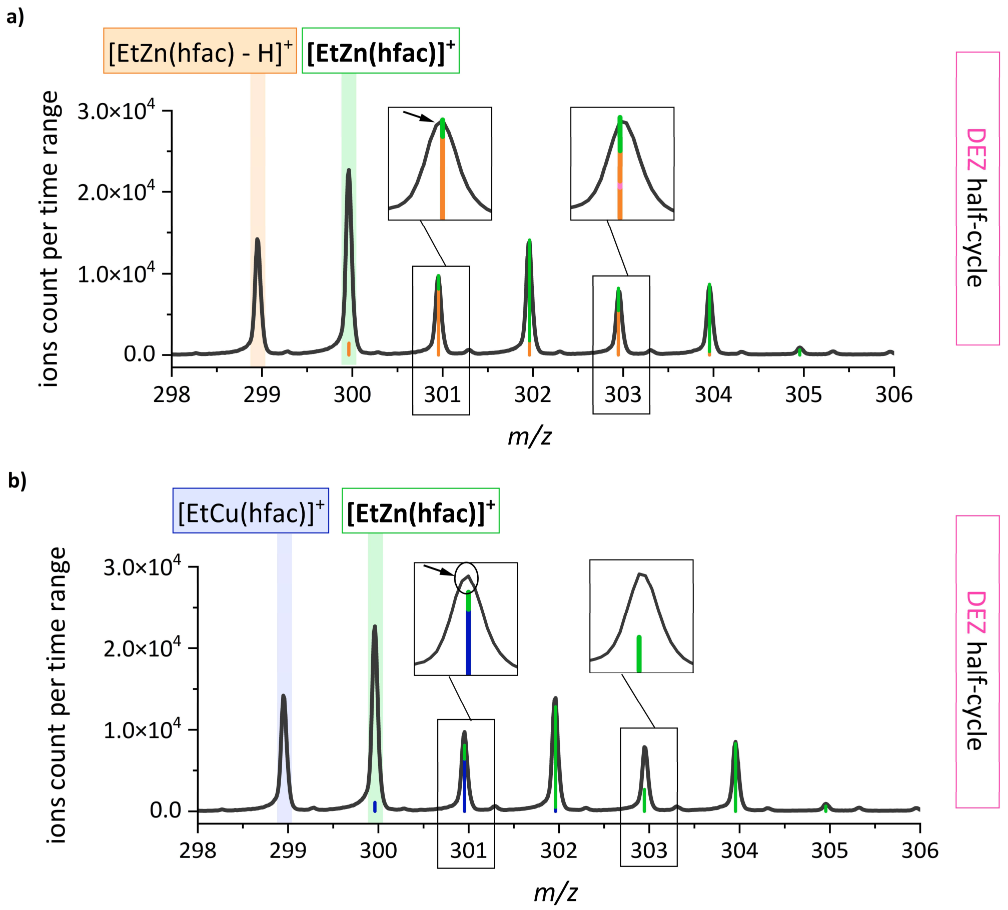

Figure S7. Isotopic distribution analysis of the *m/z* 300 fragment confirming its identification as [EtZn(hfac) – H]$^+$ rather than [EtCu(hfac)]$^+$. The observed pattern matches theoretical Zn isotopic distribution, supporting zinc role in the reaction mechanism.

S2.7 Fragment peak at *m/z* = 232

The distinct fragment peak at *m/z* 232 observed in the mass spectrum during the $Cu(hfac)_2$ half-cycle originated directly from the copper precursor fragmentation during electron ionization, not from surface reactions with DEZ, as confirmed by its presence during consecutive $Cu(hfac)_2$-only pulses (Figure S8). Additional fragments with similar isotopic signature were identified that exclude any metal-containing species. The highest mass peak was observed at *m/z* 470. Other 9 different peaks were detected, each separated by the loss of –F, –CF, or $–CF_3$ groups (as shown in the mass spectrum in Figure S8). Since these fragments are exclusively fluorinated organic compounds, we hypothesized that they originate from dimerization of the anionic ligand present in the $Cu(hfac)_2$ complex and/or from the neutral form Hhfac originated following adsorption in the ALD chamber.

Previous studies[2] have shown that the neutral hexafluoroacetylacetonate ligand undergoes dimerization upon electron impact, potentially forming a variety of dimers, including the specific structure reported in Figure S9. One such compound, with the addition of two water molecules, exhibits a parent peak with a mass-to-charge ratio of *m/z* 470. The incorporation of water may involve coordination in the hydration sphere or, most likely, directly bonding through the carbonyl group. Our data support the latter hypothesis, as no signal was observed at *m/z* 434, which would correspond to the dehydrated dimer; if the water molecules were only associated in the hydration sphere, a peak at *m/z* 434 was expected due to the weak interactions. Furthermore, all fragments identified in the mass spectrum correspond to the sequential loss of three $–CF_3$ groups and one $–CF_2$ group, matching the number of such groups in the proposed structure. However, the loss of a $–CF_2$ group at the position indicated in the structure would lead to cleavage of the dimer, and the resulting mass peaks would no longer be consistent with the observed spectrum. We therefore

tentatively propose that this compound corresponds to the structure shown, assuming a rearrangement occurs upon –CF_2 loss. Nonetheless, it is also possible that the actual structure is similar to the one in Figure S9, but with different positions for the –CF_2 and –CF_3 groups. Therefore, further analysis is required to definitively confirm the molecular structure.

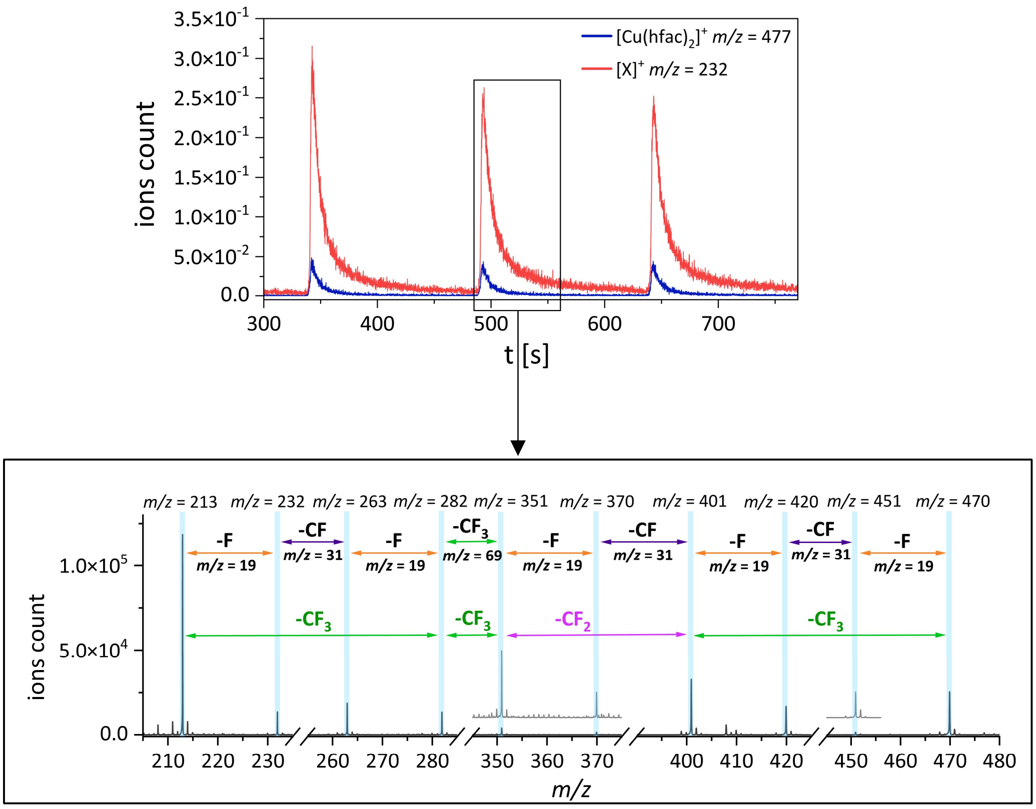

Figure S8. (top) Temporal evolution of $Cu(hfac)_2$ parent peak during $Cu(hfac)_2$-only pulses. (bottom) MS generated from the second $Cu(hfac)_2$ pulse; the MS shows the presence of several peaks, each separated one from the other for the loss of –F, –CF, or –CF_3 groups. The isotopic fingerprint of these fragments suggests that they do not contain any Zn nor Cu atoms due to the absence of a strong isotope contributions at +2 for Cu and +2 and +4 for Zn.

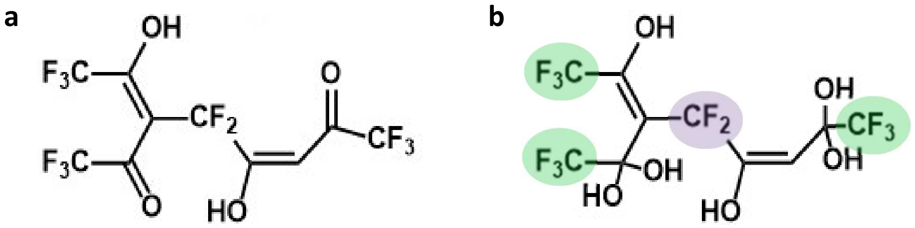

Figure S9. (a) Dimer formation upon electron impact. (b) Proposed hypothesized molecular structure for the fluorinated organic compound detected in the MS during Cu(hfac)$_2$ pulses.

S3 XAS Data Analysis

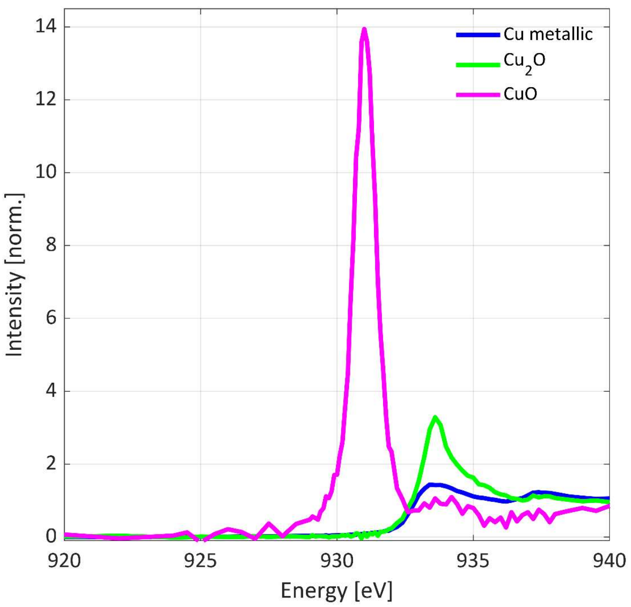

Figure S10. Cu L_3-edge reference XAS of copper components collected using Cu Lα partial fluorescence yield and normalized to edge step. These spectra were used to perform linear combination fitting shown in Figure 3a in the main article.

Based on the results of linear combination fitting of Cu L_3-edge collected by volume sensitive PFY XAS, the following speciation of the Cu phases of the film was obtained: metallic copper of approx. 85 at. %, Cu_2O of approx. 15 at. %, CuO of less than 1 at % (page 13, lines 2-4). Atomic fractions were later rescaled to volume fractions by relative volume densities of Cu atoms in both metallic Cu and Cu_2O structures, resulting in approx. volumes of 91% vs. 9%, respectively. CuO was excluded from this semi-quantitative analysis due to negligible amount of such phase. Considering that identical surface is probed for both phases, their relative volume equals the relative depth, resulting in the thickness of Cu_2O phase of 0.09×45 nm ≈ 4 nm. The assumption of the distribution of oxide phase mainly in the surface is based on common knowledge of the altering process of Cu films and is qualitatively supported by XAS results. Namely the observation

of significantly higher fraction of oxide phase in the surface-sensitive TEY XAS with respect to volume sensitive PFY XAS. As pointed out by the reviewer, TEY XAS is probing not only surface by also near surface volume of the film. In the case of Cu metal the volume is probed down to approx. 10 nm by drain current detection of TEY, which is why metal phase covered by native oxide is visible in TEY XAS.

S4 Reaction Mechanism

Table S4. Summary of theoretically possible surface reactions with solid and volatile by-products: grey - parent ion peak missing but fragmentation products visible in TOFMS, yet not unique for identification of parent molecule; bold green - most intense TOFMS signal; green - minor intense TOFMS signal; navy blue - not detected with TOFMS. Surface species are labeled with “s-” and prefixes "2." and "3." signify two and three of the entities

**Chemisorption with ZnEt$_2$**									
S’-2)	s-Cu^{0}	+	Zn^{2+}Et$_2$	→	s-Cu^{0}	+	s-Zn^{0}	+	Et$_2$
S-2)		+		→	s-Cu^{1+}-Et	+	s-Zn^{1+}-Et		
**Cu(hfac)$_2$ pulse**									
A’-1)	s-Zn^{0}	+	Cu^{2+}(hfac)$_2$	→	s-Cu^{0}	+	**Zn^{2+}(hfac)$_2$**		
A-1)	s-Zn^{1+}-Et + s-Cu^{1+}-Et	+		→	2.s-Cu^{1+}-Et	+	**Zn^{2+}(hfac)$_2$**		
				→	2.s-Cu^{0}	+	**Zn^{2+}(hfac)$_2$**	+	Et$_2$
B-1)				→	s-Cu^{1+}-hfac	+	s-Cu^{1+}-Et	+	EtZn^{2+}(hfac)
				→	2.s-Cu^{0}	+	Et(hfac)	+	EtZn^{2+}(hfac)
C-1)	2.s-Zn^{1+}-Et + 2.s-Cu^{1+}-Et	+		→	s-Cu^{0}	+	2.s-Cu^{1+}-Et	+	2.EtZn^{2+}(hfac)
				→	3.s-Cu^{0}	+	Et$_2$	+	2.EtZn^{2+}(hfac)
D-1)	s-Zn^{1+}-Et + s-Cu^{1+}-Et	+		→	s-Zn^{1+}-hfac	+	s-Cu^{1+}-Et	+	EtCu^{2+}(hfac)
				→	s-Zn^{0} + s-Cu^{0}	+	Et(hfac)	+	EtCu^{2+}(hfac)
E-1)	2.s-Zn^{1+}-Et + 2.s-Cu^{1+}-Et	+		→	2.s-Zn^{1+}-Et	+	s-Cu^{0}	+	2.EtCu^{2+}(hfac)
				→	2.s-Zn^{0} + s-Cu^{0}	+	Et$_2$	+	2.EtCu^{2+}(hfac)
**Chemisorption with Cu(hfac)$_2$**									
S-1)	s-Cu^{0}	+	Cu^{2+}(hfac)$_2$	→	s-Cu^{1+}-hfac	+	s-Cu^{1+}-hfac		
**ZnEt$_2$ pulse**									
A-2)	2.s-Cu^{1+}-hfac	+	Zn^{2+}Et$_2$	→	2.s-Cu^{1+}-Et	+	Zn^{2+}(hfac)$_2$		
				→	2.s-Cu^{0}	+	Zn^{2+}(hfac)$_2$	+	Et$_2$
B-2)				→	s-Cu^{1+}-hfac	+	s-Cu^{1+}-Et		**EtZn^{2+}(hfac)**
				→	2.s-Cu^{0}	+	Et(hfac)	+	**EtZn^{2+}(hfac)**
C-2)			2.Zn^{2+}Et$_2$	→	2.s-Cu^{1+}-Et	+	**2.EtZn^{2+}(hfac)**		
				→	2.s-Cu^{0}	+	**2.EtZn^{2+}(hfac)**	+	Et$_2$
D-2)			Zn^{2+}Et$_2$	→	s-Cu^{1+}-hfac	+	s-Zn^{1+}-Et	+	EtCu^{2+}(hfac)
				→	s-Cu^{0} + s-Zn^{0}	+	Et(hfac)	+	EtCu^{2+}(hfac)
E-2)			2.Zn^{2+}Et$_2$	→	2.s-Zn^{1+}-Et	+	2.EtCu^{2+}(hfac)		
				→	2.s-Zn^{0}	+	2.EtCu^{2+}(hfac)	+	Et$_2$

REFERENCES

(1) Hornsveld, N.; Kessels, W. M. M.; Creatore, M. Mass Spectrometry Study of Li 2 CO 3 Film Growth by Thermal and Plasma-Assisted Atomic Layer Deposition. *Journal of Physical Chemistry C* **2019**, *123* (7), 4109–4115.

(2) Kholodkova, E. M.; Ponomarev, A. V. Radiolytic Transformations of Liquid Hexafluoroacetylacetone. *Radiation Physics and Chemistry* **2024**, *217*, 111453.
